# IFITM3 enhances immunosensitivity via MHC-I regulation and is associated with the efficacy of anti-PD-1/-L1 therapy in SCLC

**DOI:** 10.1186/s12943-025-02383-x

**Published:** 2025-07-03

**Authors:** Yanan Cui, Tianyu Qiu, Jiale Wang, Xinyu Liu, Libo Luo, Jizhong Yin, Xinxin Zhi, Wanying Wang, Gaohua Feng, Chunyan Wu, Zhikai Zhao, Hua Zhang, Fei Li, Fengying Wu, Shengxiang Ren

**Affiliations:** 1 Department of Medical Oncology, Shanghai Pulmonary Hospital, Tongji University School of Medicine, Shanghai, 200433 China; 2 Department of Pulmonary and Critical Care Medicine, Zhangjiagang Hospital of Traditional Chinese Medicine, Suzhou, China; 3 Department of Pathology, Shanghai Pulmonary Hospital, Tongji University School of Medicine, Shanghai, 200433 China; 4 Hillman Cancer Center, UPMC, Pittsburgh, PA 15232 USA; 5 Department of Medicine, Division of Hematology/Oncology, University of Pittsburgh School of Medicine, Pittsburgh, PA 15261 USA; 6 Department of Pathology, Frontier Innovation Center, School of Basic Medical Sciences, Fudan University, Shanghai, 200032 China

**Keywords:** Small cell lung cancer, Immunotherapy, MHC-I, IFITM3

## Abstract

**Background:**

Most small cell lung cancer (SCLC) patients exhibit resistance to immune checkpoint inhibitors (ICIs) and demonstrate downregulation of major histocompatibility complex class I (MHC-I) molecules. This study aimed to elucidate the regulatory mechanisms underlying MHC-I expression and potential combination strategies.

**Methods:**

Single-cell and bulk RNA sequencing data from SCLC patients were analyzed. Clinical data from SCLC patients treated with PD-1/PD-L1 inhibitors were used to investigate the associations between treatment efficacy and IFITM3 expression. In vitro and in vivo functional studies were conducted to evaluate the role and mechanisms of IFITM3 in modulating tumor sensitivity to PD-1 inhibitors.

**Results:**

Integrative analysis of multiple real-world SCLC cohorts confirmed a significant positive association between IFITM3 expression and MHCI. IFITM3 overexpression upregulated MHC-I-related genes, enriched antigen presentation pathways, and increased CD8^+^ T-cell infiltration and cytotoxicity. Elevated IFITM3 expression was significantly associated with prolonged progression-free survival (PFS) in patients receiving chemoimmunotherapy but not in those treated with chemotherapy alone. Additionally, patients with high H-scores for IFITM3, as determined by immunohistochemistry, demonstrated better clinical outcomes with chemoimmunotherapy. Inducing IFITM3 expression directly or through treatment with ethyl gallate (EG), an IFITM3 inducer, effectively sensitized tumors to PD-1 blockade in SCLC mouse models. Mechanistic studies revealed that IFITM3 upregulates NLRC5, a key transcriptional activator of MHC-I, facilitating its nuclear translocation and thereby increasing MHC-I levels.

**Conclusions:**

IFITM3 is associated with MHC-I expression and can predict the efficacy of anti-PD-1/-L1 therapy in SCLC patients. IFITM3 inducers potently improved the efficacy of anti-PD1 monotherapy in SCLC.

**Supplementary Information:**

The online version contains supplementary material available at 10.1186/s12943-025-02383-x.

## Background

Immune checkpoint inhibitors (ICIs), particularly PD-1/PD-L1 inhibitors combined with chemotherapy, have become the front-line standard of care for extensive-stage small cell lung cancer (SCLC). However, fewer than 20% of patients achieve substantial and durable responses, underscoring the critical need for reliable predictive biomarkers to identify individuals most likely to benefit. Novel combinational therapeutic strategies are urgently needed to improve the efficacy of immunotherapy [1, 2].

PD-L1 expression and the tumor mutational burden (TMB) have been established as predictive biomarkers for immunotherapy in various solid tumors. However, their predictive value is invalid in the context of chemoimmunotherapy for extensive-stage SCLC [3]. Instead, the expression of major histocompatibility complex class I (MHC-I) has emerged as an important predictive biomarker for SCLC [4–7]. MHC-I is critical for presenting tumor antigens to cytotoxic T cells, thus enabling effective antitumor immune responses [8]. However, SCLC is inherently poorly immunogenic and is characterized by intrinsically low MHC I expression [3]. Recent evidence suggests that this deficiency is partly driven by epigenetic programming rather than genetic mutations, positioning the restoration of MHC-I expression as a key strategy for SCLC treatment. For example, PRC2-mediated silencing of MHC-I antigen processing and presentation genes can be alleviated through the pharmacological inhibition of polycomb repressive complex 2 (PRC2) components, including Menin and EZH2 inhibitors, which have been shown to restore MHC-I expression in SCLC cell lines and enhance durable responses to ICIs [4, 9, 10]. In addition, the inhibition of lysine-specific demethylase 1 (LSD1) with compounds such as bomedemstat has been reported to enhance MHC-I-mediated antigen presentation, sensitizing SCLC tumors to PD-1/PD-L1 inhibitors [11, 12]. Despite these advances, other regulatory mechanisms governing MHC-I expression remain underexplored, highlighting the need for further research in this area.

Recent studies have emphasized the remarkable plasticity of SCLC subtypes, demonstrating their ability to transition between distinct molecular states, which drives intratumoral heterogeneity (ITH) [13, 14]. To identify novel MHC-I regulators in SCLC, we analyzed single-cell RNA sequencing (scRNA-seq) data from a real-world cohort [15]. Our analysis revealed the presence of distinct tumor cell subpopulations with varying MHC-I expression within the same tumor. Through pseudotime analysis of tumor cells from scRNA-seq data, we identified molecular changes linked to differential MHC-I expression and established interferon-induced transmembrane protein 3 (IFITM3) as a key regulator of MHC-I in SCLC. By integrating clinical data analysis with in vitro and in vivo experiments, we established IFITM3 as a promising biomarker for predicting the immunotherapy response in SCLC patients and proposed a novel therapeutic strategy to improve clinical outcomes.

## Methods

### Analysis of scRNA-seq data from SCLC

ScRNA-seq data were obtained from nine treatment-naive SCLC tumor samples [15]. Tumor samples were collected via diagnostic procedures, including transcutaneous needle biopsy and bronchoscopy. After quality control, 12,897 cells were retained for analysis, and 16 distinct clusters were identified. The cancer cell cluster was characterized by the absence of normal lung epithelial markers and the presence of EPCAM, which was further validated via InferCNV analysis. In total, 11,718 cancer cells were reclustered via the Seurat (v4.0) R package. Clustering was performed via the Louvain algorithm at a resolution of 0.5, and dimensionality reduction was visualized via uniform manifold approximation and projection (UMAP).

MHC-I-related gene activity was quantified via AUCell, which computes per-cell activity scores on the basis of the area under the curve (AUC) for the gene set. Additional methods, including UCell and a single score, were employed to generate per-cell enrichment scores on the basis of gene expression ranks to assess MHC-I activity comprehensively.

Pseudotime trajectory analysis was conducted via the Monocle R package, with UMAP embeddings visualizing cell progression along pseudotime. This analysis highlights cellular transitions and potential lineage differentiation. Seurat objects were converted into monocle-compatible datasets for unsupervised identification of genes associated with developmental divergence. Gene Ontology (GO) enrichment analysis, performed with the “Enrichr” package, identified significant GO terms with an adjusted p value threshold of < 0.05.

### Analysis of public bulk RNA-seq data

Bulk RNA-seq data for SCLC were obtained from publicly available datasets, including the GSE99316, GSE40275, and GSE60052 datasets from the Gene Expression Omnibus (GEO), Cancer Cell Line Encyclopedia (CCLE), and sclc_ucologne datasets from cBioPortal for Cancer Genomics. Pan-cancer datasets from CCLE and TCGA were obtained from the cBioPortal for Cancer Genomics. Spearman’s correlation analysis was conducted to identify significant correlations, defined by an absolute correlation coefficient (|r|) > 0.5 and a p value < 0.05. Immune phenotypes (IPS) were calculated via the “IOBR” R package [16], and immune and stromal cell populations were estimated via the “MCPcounter” R package.

RNA-seq data and clinical information for the Impower133 cohort were obtained from the European Genome-phenome Archive (EGA), whereas data for the PH cohort, comprising extensive-stage SCLC patients receiving first-line chemoimmunotherapy, were collected as part of a phase II clinical study conducted by our group at Shanghai Pulmonary Hospital [17]. Data for the Rochester cohort were sourced from a previously published study [18]. Progression-free survival (PFS) was defined as the time from treatment initiation to either radiographic or clinical disease progression, or death from any cause, whichever occurred first. Patients without progression at the time of last follow-up were censored. PFS analysis was performed using the R packages “survival” and “survminer”. Optimal gene expression cutoffs were determined using the maximally selected rank statistic (maxstat) implemented in the “survminer” package. Kaplan-Meier survival curves were generated to compare outcomes between groups, and statistical significance was assessed using the log-rank test. Hazard ratios (HRs) and 95% confidence intervals (CIs) were calculated via Cox proportional hazard models.

### Clinical sample collection and immunohistochemistry (IHC)

Clinical data and formalin-fixed paraffin-embedded (FFPE) tissue samples were collected from 42 patients diagnosed with extensive-stage SCLC at the Shanghai Pulmonary Hospital (Supplementary Table 1). All patients received first-line chemotherapy combined with ICIs as part of standard care. Treatment response was evaluated according to the Response Evaluation Criteria in Solid Tumors (RECIST) version 1.1. Objective response rate (ORR) was calculated as the proportion of patients who achieved a complete response (CR) or partial response (PR).

For IHC analysis, FFPE sections were deparaffinized, rehydrated, and subjected to antigen retrieval. Primary antibodies against IFITM3 (Proteintech Cat# 11714-1-AP), CD8 (Proteintech Cat# 15240-1-AP), and MHC-I (Proteintech Cat# 15240-1-AP, Cat# 66013-1-Ig) were used, followed by incubation with biotin-conjugated secondary antibodies and avidin-biotin-peroxidase complexes. Diaminobenzidine (DAB) served as the chromogen to visualize the target proteins, producing a brown precipitate at the antigen sites. The sections were counterstained with hematoxylin, scanned, and analyzed via 3D HISTECH QuantCenter 2.1 software. H-scores were used to quantify the immunohistochemical results and were calculated via the following formula: H-score =∑(pi×i) = (percentage of weak intensity ×1) + (percentage of moderate intensity ×2) + (percentage of strong intensity ×3) [19].

### Cell culture

Four human SCLC cell lines, NCI-H69, NCI-H446 were obtained from the Institute of Biochemistry and Cell Biology of the Chinese Academy of Sciences (Shanghai, China). NCI-H82 and NCI-H526 cell lines were obtained from the American Type Culture Collection (ATCC, Manassas, VA, USA). The cells were cultured in RPMI-1640 medium supplemented with 10% fetal bovine serum (FBS) and 1% penicillin‒streptomycin (100 U/mL penicillin and 100 µg/mL streptomycin). The murine SCLC cell line RPP631, derived from a syngeneic genetically engineered mouse model (GEMM) with biallelic loss of Trp53 and Rb1 as well as deletion of the Rb family member p130 (RPP), was maintained in HITES medium [20]. All the cell lines were cultured at 37 °C in a humidified atmosphere containing 5% CO₂ to ensure optimal growth conditions.

### In vitro drug treatment

NCI-H446 and NCI-H69 cells were treated with dexamethasone (Sigma Aldrich, Cat#D4902)) (1 µM and 10 µM) or cisplatin (Accord Healthcare, Cat#16729-288-11) (5 µM and 10 µM) for 24–48 h. Cells were then harvested for Western blot analysis.

### Plasmid DNA and SiRNA transfection

Plasmids encoding the open reading frame (ORF) of human *IFITM3* (Genecopeia, Cat#A3792) and mouse *Ifitm3* (Genecopeia, Cat#Mm08357) were used for overexpression experiments. Small interfering RNAs (siRNAs) targeting *IFITM3* were purchased from RiboBio (Guangzhou, China). The sequences of the siRNA are listed in Supplementary Table 2. Plasmids encoding HA-tagged *IFITM3* (Cat# 58397; Addgene) and MYC-tagged *NLRC5* (Cat# 37509; Addgene) were used. Transfection was performed via Lipofectamine 3000 (Invitrogen) according to the manufacturer’s instructions. The cells were harvested 48 h posttransfection for downstream analysis via Reverse Transcription quantitative Polymerase Chain Reaction (RT-qPCR) and western blotting.

### RNA Preparation and RT-qPCR

Total RNA was extracted via an RNA Rapid Extraction Kit (Cat# 220011; Feijie Bio) following the manufacturer’s protocol. Reverse transcription of RNA into complementary DNA (cDNA) was performed via the PrimeScript RT Reagent Kit (Takara, Cat#RR036A). RT-qPCR was performed using SYBR Green Master Mix (Takara, Cat# RR820A) to measure the gene expression levels. The delta‒delta CT (2^-ΔΔCT) method was used to analyze the relative expression, with GAPDH used as the internal normalization control. The sequences of primers used for RT-qPCR are listed in Supplementary Table 2.

### Western blot assays

Cell lysates were prepared via radioimmunoprecipitation assay (RIPA) buffer and subjected to sodium dodecyl sulfate-polyacrylamide gel electrophoresis (SDS-PAGE) for protein separation. Proteins were then transferred onto PVDF membranes (Millipore Sigma, Cat# IPVH00010). The membranes were blocked with blocking buffer and incubated with primary antibodies specific to the target proteins. The following antibodies were used: GAPDH (Proteintech Cat# 60004-1-Ig) as an internal control, IFITM3 (Proteintech Cat# 11714-1-AP; Abcam Cat#ab15592), and MHC-I (Proteintech Cat# 15240-1-AP, Cat# 66013-1-Ig; Cell Signaling Technology Cat#35923). After washing, the membranes were incubated with appropriate horseradish peroxidase-conjugated secondary antibodies. The protein bands were visualized via an enhanced chemiluminescence (ECL) detection system and imaged via autoradiography.

### Lentiviral packaging and transduction of SCLC cells

Lentiviral particles were generated by transfecting HEK293T cells with *IFITM3* shRNA or overexpression plasmids using a lentiviral packaging system. Culture supernatants from HEK293T cells containing lentiviral particles were used to transduce SCLC cells in the presence of 8 µg/mL polybrene (Cat#ST1380; Beyotime). The cells were centrifuged with the viral supernatant at 2,000 rpm for 45 min, followed by incubation at 37 °C in a humidified atmosphere with 5% CO₂. After 6 h, the medium was replaced with HITES medium, and virus-infected cells were selected with 1.0 µg/mL puromycin (Beyotime, Cat#ST551) starting 48 h post infection. The shRNA sequences targeting IFITM3 used for lentiviral transduction are listed in Supplementary Table 2.

### Animal model and treatment

Six-week-old male BALB/c nude and C57BL/6 mice were obtained from Slake (Shanghai, China) and housed in accredited pathogen-free facilities. The mice were acclimated for one week at the Animal Facility of Shanghai Pulmonary Hospital prior to the experiments. For subcutaneous tumor implantation, 2 × 10⁶ RPP cells (with control or IFITM3 overexpression) were resuspended in a 1:1 mixture of PBS and Matrigel (Beyotime, Cat# C0383) and injected subcutaneously into the mice. Treatment was initiated when the average tumor volume reached approximately 100 mm³. The mice were randomized into treatment groups and treated with either 0.85% saline (vehicle control) or the anti-PD-1 antibody. Anti-mouse PD-1 (HRP00262-012, provided by Hengrui Medicine Co., Jiangsu, China) was administered intraperitoneally at 10 mg/kg three times per week for three weeks. Tumor volumes were measured every 2–3 days via calipers and were calculated as follows: volume = 1/2×(length×width^2^).

For the ethyl gallate (EG) experiments, RPP cells were injected subcutaneously into C57BL/6 mice. The mice were randomized into four groups: vehicle control (5% DMSO in 10% Tween 80), anti-PD-1 antibody alone, EG alone (100 mg/kg/day, administered orally by gavage), and a combination of anti-PD-1 and EG. Tumor growth and body weight were regularly monitored.

### Flow cytometry

For human SCLC samples, the cells were blocked with TruStain FcX (BioLegend, Cat# 422301) to prevent nonspecific binding and stained at 4 °C for 30 min with an anti-human HLA-ABC antibody (BD Biosciences Cat# 555553) at the appropriate dilution. According to the manufacturer’s instructions, murine tumor samples were dissociated into single-cell suspensions via a tumor dissociation kit (Miltenyi, Cat#130-096-730). The cells were stimulated for 4 h for intracellular cytokine staining with a leukocyte activation cocktail (BD Biosciences Cat# 550583). Single-cell suspensions were blocked with anti-CD16/CD32 (BD Biosciences Cat# 553141) and stained with the following anti-mouse antibodies: CD45 (BD Biosciences Cat# 557659), CD3 (BD Biosciences Cat# 551163), CD4 (BD Biosciences Cat# 563151), CD8 (BD Biosciences Cat# 557959), GZMB (Thermo Fisher Scientific Cat# 12-8898-82), CD69 (BD Biosciences Cat# 552879), CD44 (BD Biosciences Cat# 563970), and live/dead (BD Biosciences Cat# 564406). Stained cells were acquired on a Beckman CytoFLEX flow cytometer, and data analysis was performed via FlowJo software.

### RNA sequencing and analysis

RNA extraction and sequencing were performed by Berry Genomics Co. Ltd. (Beijing, China). Transcript counts and abundances were quantified from RNA-seq reads via Salmon version 1.1.0 [21] and aligned to the 25-mer indexed human reference genome (hg38). Transcript-to-gene mapping was conducted via Ensembl 92 annotations [22], and expression levels were normalized at the gene level via size factors. Principal component analysis (PCA) and differential expression analysis were performed using the R package DESeq2, with significantly differentially expressed genes (DEGs) defined as those with an absolute log2(fold change) > 1 and *p* < 0.05. Gene set enrichment analysis (GSEA) was carried out using the R package GSVA, and normalized enrichment scores (NES) were calculated. Interferon-stimulated genes (ISGs) were defined as genes included in the HALLMARK INTERFERON ALPHA RESPONSE and HALLMARK INTERFERON GAMMA RESPONSE gene sets from the Molecular Signatures Database.

### Subcellular fractionation assays

Subcellular fractionation was performed via the Subcellular Protein Fractionation Kit for Cultured Cells (Cat#78840; Thermo Fisher Scientific) according to the manufacturer’s instructions. Briefly, the cells were harvested and washed with ice-cold PBS prior to lysis. Sequential extraction was performed to isolate the cytoplasmic, membrane, and nuclear fractions. The cell pellets were initially incubated with cytoplasmic extraction buffer and centrifuged to obtain the cytoplasmic fraction. The remaining pellet was treated with membrane extraction buffer to extract the membrane fraction. Finally, the nuclear fraction was obtained by treating the residual pellet with Nuclear Extraction Buffer, followed by centrifugation. Protein concentrations were determined via a BCA protein assay kit (Cat# A55865; Thermo Fisher), and equal amounts of protein were used for western blot analysis.

### Coimmunoprecipitation (Co-IP)

Dynabeads A and G (25 µL each; Thermo Fisher, Cat#10003D, #10001D) were mixed, washed twice with PBS containing 5 mg/mL bovine serum albumin (BSA), and resuspended in 600 µL of PBS + BSA. Antibodies (4 µg) or IgG controls were added, and the mixture was incubated at 4 °C with rotation for 5–6 h. Approximately 10 million cells were harvested, washed with PBS, and lysed in NP40 lysis buffer (Thermo Fisher, Cat# J60766. AK), supplemented with protease inhibitors. The cell lysates were sonicated, followed by centrifugation at 13,500 RPM for 10 min. Following antibody-bead incubation, the beads were washed with NP40 lysis buffer and resuspended in sonicated lysates. The mixture was then incubated overnight at 4 °C with rotation. The beads were washed five times with NP40 buffer, and the proteins were prepared for western blot analysis by boiling in LDS sample buffer.

### Immunofluorescence staining

The coverslips were coated with 50 µg/mL working solution of poly-D-lysine (Gibco, Cat# A3890401), and the cells were seeded onto the coated surface. The cells were fixed with 4% PFA in PBS for 10 min at room temperature in the dark, followed by three washes with PBS. Permeabilization was performed via the addition of 0.25% Triton X-100 in PBS for 5 min, followed by two additional PBS washes. Blocking was performed with 5% BSA in PBS for 1 h at room temperature. Primary antibodies diluted in 1% BSA were applied to the cells, which were subsequently incubated overnight at 4 °C in a light-protected container. Following three washes with PBS, secondary antibodies diluted in 0.5% BSA were applied and incubated in the dark for 60 min. The cells were washed three times with PBS, incubated with DAPI for 5 min, and mounted with mounting medium (DAKO, Cat #S3023). The antibodies used were HA-Tag (Cell Signaling Technology Cat# 3724), MYC-Tag (Cell Signaling Technology Cat# 2276), goat anti-rabbit secondary antibody (Thermo Fisher Scientific Cat# A-21245), and goat anti-mouse secondary antibody (Thermo Fisher Scientific Cat# A-11001).

### Statistics

All the statistical analyses were performed via IBM SPSS version 24 and R version 3.5.1. The two groups were compared via two-tailed Student’s t test or the Wilcoxon rank-sum test. All p values were two-sided, with a significance threshold of *P* < 0.05.

## Results

### Integrative analysis of multiple real-world SCLC cohorts identifies IFITM3 as a key potential regulator of MHC-I

**Fig. 1:**
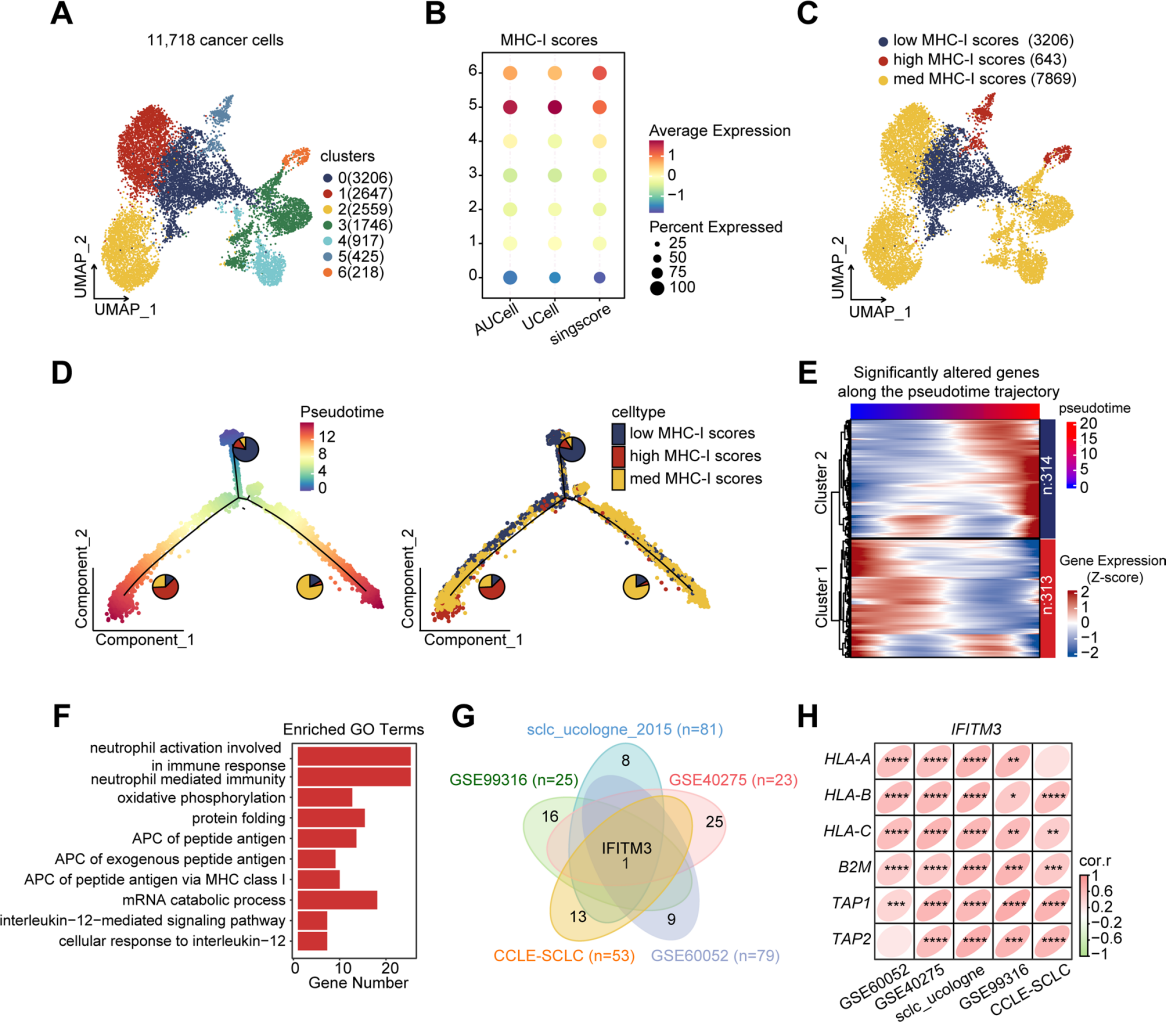
IFITM3 is correlated with MHC-I expression in SCLC. **(A)** UMAP plot displaying 11,718 cancer cells from single-cell RNA sequencing of nine untreated SCLC tumors. **(B)** Dot plot representing MHC-I gene set scores calculated via the AUCell, UCell, and singscore algorithms. The dot size indicates the percentage of cells expressing the MHC-I gene set, and the color represents the average expression level. **(C)** UMAP plot categorizing cancer cells on the basis of MHC-I expression levels. **(D)** Pseudotime trajectory initiated from MHC-I low clusters toward MHC-I high clusters. Pie charts represent the proportion of cells within each category along the trajectory. **(E)** Heatmap showing 627 genes whose expression significantly changed along the pseudotime trajectory. Cells were ordered by pseudotime (left to right), with gene expression scaled by Z-score (bottom color bar). Clusters represent distinct temporal gene expression patterns: Cluster 1 genes were enriched in early pseudotime states (low-MHC-I cells), and Cluster 2 genes in late pseudotime states (high-MHC-I cells). Sample size: *n* = 11,718 cells from 9 patients. **(F)** Bar plot of enriched GO terms for the Cluster 2 gene set from (E). **(G)** Venn diagram showing overlapping MHC-I-related genes (correlation *r* > 0.5, *P* < 0.05) across five SCLC datasets. **(H)** Correlation matrix depicting *IFITM3* expression and key MHC-I genes across the five datasets. Spearman correlation coefficients were calculated for each gene pair. *P < 0.05, **P < 0.01, ***P < 0.001, ****P < 0.0001

To identify new genes associated with MHC-I levels in SCLC, we analyzed scRNA-seq data from nine untreated SCLC patients [15], characterizing transcriptomic variations in cancer cells with different MHC-I levels. Seven distinct subpopulations of 11,718 cancer cells were identified (Fig. 1A). MHC-I scores within these subgroups were quantified via three independent algorithms (AUCell, UCell, and single score) on the basis of an established MHC-I-related gene set (*TAP1*, *TAP2*, *B2M*, *HLA-A*, *HLA-B*, and *HLA-C*) (Fig. 1B) [6]. Subpopulations 5 and 6 were categorized into high MHC-I score clusters. In contrast, subgroup 0 was classified into low MHC-I score clusters. Subgroups 1, 2, 3, and 4 were defined as intermediate MHC-I clusters (Fig. 1C).

Tumor heterogeneity analysis revealed that most patients exhibited intratumoral variability in MHC-I expression. Notably, tumor cells from patient P03 were predominantly derived from cluster 5, which was characterized by high MHC-I expression, while cells from patient P07 were mainly distributed within cluster 0, exhibiting low MHC-I expression (Supplementary Fig. 1A). Based on established molecular subtyping of SCLC [23], P03 was classified as the NE-I subtype, previously reported to be more responsive to immunotherapy, whereas P07 belonged to the SCLC-A subtype, which has been associated with immunotherapy resistance (Supplementary Fig. 1B). These findings underscored the potential of MHC-I expression levels to stratify patients and predict immunotherapeutic outcomes.

Pseudotime analysis was conducted to model the differentiation trajectory of cancer cells transitioning from low to high MHC-I expression. This revealed two differentiation pathways: one transitioning toward intermediate MHC-I clusters and the other toward high MHC-I clusters (Fig. 1D), indicating a progressive increase in MHC-I levels. Unsupervised clustering of genes whose expression significantly changed along the pseudotime trajectory revealed two gene sets, Cluster 1 and Cluster 2, that were enriched at the beginning and end of the trajectory, respectively (Fig. 1E). Genes from the Cluster 2 set that were highly expressed in high-MHC-I clusters were further analyzed for their potential to induce MHC-I expression and enhance sensitivity to immunotherapy. GO analysis indicated that these genes were enriched in processes such as MHC class I protein complex assembly and antigen processing and presentation (Fig. 1F).

To identify the key molecular factors associated with MHC-I expression, correlations between Cluster 2 genes and MHC-I levels were analyzed across multiple human SCLC datasets (sclc_ucologne_2015, GSE99316, GSE40275, GSE60052, and CCLE) [24–27]. Spearman correlation analysis revealed *IFITM3* as the only gene that was consistently and significantly correlated with MHC-I expression across cohorts (Spearman *r* > 0.5, *P* < 0.05) (Fig. 1G-H). This correlation was further validated via the CCLE protein dataset, which revealed a positive correlation between IFITM3 and MHC-I-related genes at the protein level (Supplementary Fig. 2A). In support of these findings, single-cell RNA-seq analysis showed a gradual increase in *IFITM3* expression along the pseudotime differentiation trajectory, aligning with the progressive upregulation of MHC-I (Supplementary Fig. 2B). Moreover, UMAP visualization confirmed that tumor cell clusters with high MHC-I expression exhibited concomitantly elevated *IFITM3* levels (Supplementary Fig. 2C). Collectively, these results suggest that IFITM3 may play a central role in modulating MHC-I expression in SCLC.

Furthermore, analysis of the Broad CCLE database revealed significantly lower IFITM3 expression in SCLC compared to tumor types characterized by higher MHC-I expression (Supplementary Fig. 2D). This pattern was further confirmed in an independent dataset comprising 21 SCLC samples, 24 lung adenocarcinoma (LUAD) samples, and four normal lung tissues, which consistently showed reduced IFITM3 expression in SCLC (Supplementary Fig. 2E). These findings further underscore the relevance of IFITM3 downregulation in SCLC and support its potential role in MHC-I suppression.

To explore the broader relevance of this association beyond SCLC, we performed pan-cancer analyses and found that *IFITM3* expression was consistently positively correlated with key MHC-I genes in tumors characterized by low baseline MHC-I expression, including neuroblastoma, prostate cancer, low-grade gliomas, and breast cancer [28–31] (Supplementary Figs. 2F). We further calculated Spearman correlations between *IFITM3* expression and MHC-I scores across cancer types and observed a consistent inverse relationship between the average MHC-I score and the strength of this correlation (Supplementary Figs. 2G-H). Tumors with lower baseline MHC-I levels exhibited stronger *IFITM3*-MHC-I associations, suggesting that IFITM3 may play a more prominent regulatory role in immunologically “cold” tumors with intrinsically low MHC-I expression.

### IFITM3 upregulates MHC-I expression in SCLC

**Fig. 2:**
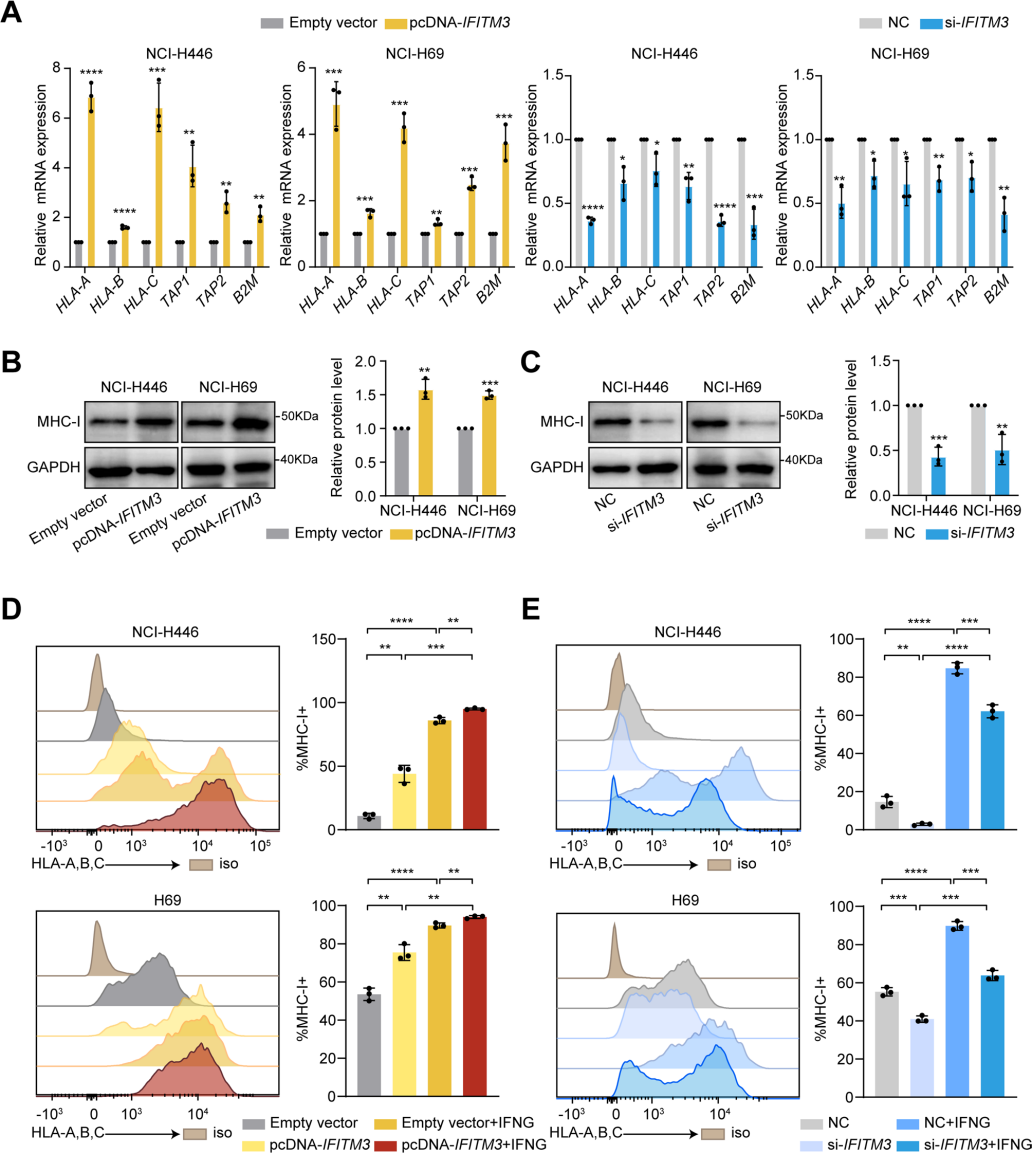
IFITM3 regulates MHC-I expression in SCLC cell lines. **(A)** Relative mRNA expression of MHC-I-related genes (*HLA-A*, *HLA-B*, *HLA-C*, *TAP1*, *TAP2*, and *B2M*) in NCI-H446 and NCI-H69 cells after overexpression or knockdown of *IFITM3*. **(B-C)** Western blot analysis of MHC-I protein levels in NCI-H446 and NCI-H69 cells after overexpression or knockdown of *IFITM3*. **(D-E)** Flow cytometry analysis of MHC-I surface expression in NCI-H446 and NCI-H69 cells after overexpression or knockdown of *IFITM3*, with or without IFN-γ treatment. The results are presented as the means ± SEMs, *n* = 3. Statistical comparisons were performed using unpaired two-tailed Student’s t-tests. *P < 0.05, **P < 0.01, ***P < 0.001, ****P < 0.0001

To assess whether the relationship between IFITM3 and MHC-I expression is correlative or regulatory, we selected SCLC cell lines with varying levels of IFITM3 and MHC-I expression based on CCLE-SCLC RNA-seq data (Supplementary Fig. 3A) and a previous study [11]. NCI-H446 and NCI-H69, which exhibit intermediate IFITM3 and MHC-I expression levels, were used as primary models for IFITM3 overexpression and knockdown (Supplementary Fig. 3B-C). RT-qPCR analysis showed that MHC-I-associated genes (*TAP1*, *TAP2*, *B2M*, *HLA-A*, *HLA-B*, and *HLA-C*) were upregulated following *IFITM3* overexpression and downregulated upon *IFITM3* knockdown in both cell lines (Fig. 2A). Western blot analysis further confirmed that *IFITM3* overexpression markedly increased MHC-I protein levels, whereas *IFITM3* knockdown led to a pronounced decrease, with consistent trends observed in both NCI-H446 and NCI-H69 cells (Fig. 2B-C). To further validate these findings, we examined two additional SCLC cell lines. In NCI-H526, which expresses high endogenous levels of MHC-I, *IFITM3* knockdown significantly reduced both MHC-I-related gene expression (Supplementary Fig. 3D) and total MHC-I protein levels (Supplementary Fig. 3E). Conversely, in NCI-H82 cells, which have very low baseline MHC-I expression, *IFITM3* overexpression induced upregulation of MHC-I-related genes and elevated MHC-I protein expression (Supplementary Fig. 3F-G).

Given that IFITM3 is an ISG and that low-dose IFN-γ has been reported to induce MHC-I expression in SCLC cell lines [11], we examined whether IFN-γ stimulation also upregulates IFITM3 expression in SCLC. IFN-γ significantly increased *IFITM3* expression in NCI-H446 and NCI-H69 cells (Supplementary Fig. 3H). To further delineate the role of IFITM3 in IFN-γ-induced MHC-I expression, we compared the MHC-I reactivation levels following *IFITM3* overexpression with those induced by IFN-γ treatment alone or in combination. *IFITM3* overexpression restored MHC-I expression to levels comparable to those of IFN-γ treatment alone, whereas combined *IFITM3* overexpression and IFN-γ stimulation further increased MHC-I expression in both cell lines (Fig. 2D). In contrast, knockdown of *IFITM3* significantly suppressed MHC-I expression, even in the presence of IFN-γ stimulation (Fig. 2E). These findings demonstrated that IFITM3 is a key regulator of MHC-I expression in SCLC and plays a critical role in IFN-γ-induced MHC-I upregulation.

### IFITM3 serves as a potential biomarker in SCLC immunotherapy

**Fig. 3:**
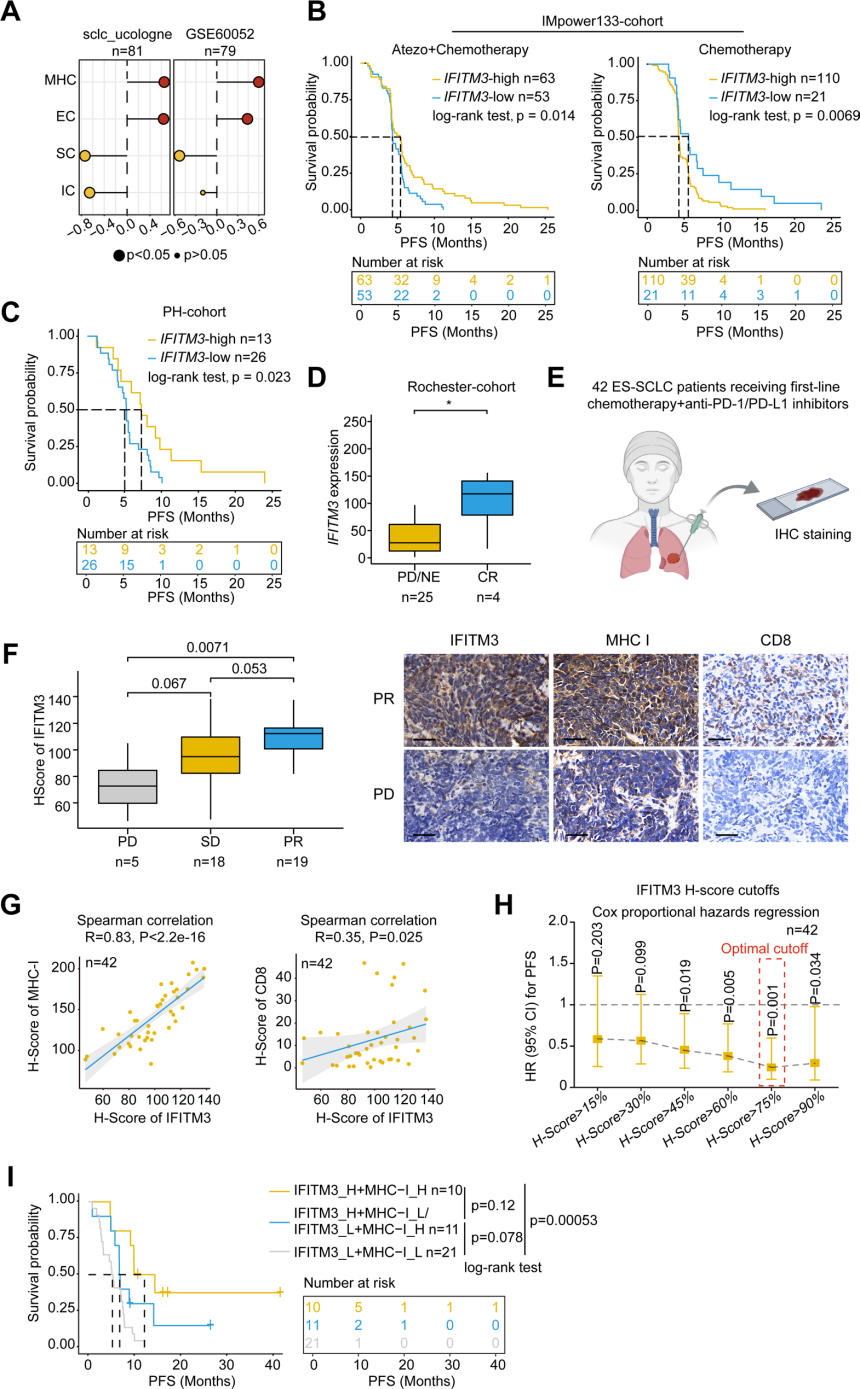
Clinical correlation of IFITM3 expression with immunotherapy outcomes in SCLC. **(A)** Scatter plot showing the correlation between *IFITM3* expression and immune-related parameters via the IPS algorithm. The x-axis represents the spearman correlation coefficient. **(B)** Kaplan-Meier curves of PFS for SCLC patients from the IMpower133 cohort treated with anti-PD-L1 plus chemotherapy (left) or chemotherapy alone (right), stratified by the optimal *IFITM3* expression cutoff. Statistical significance was assessed using the log-rank test. **(C)** Kaplan-Meier survival curve for PFS in the PH cohort stratified by the optimal *IFITM3* expression cutoff. Statistical significance was assessed using the log-rank test. **(D)** Box plot comparing *IFITM3* expression between patients with PD/NE and patients with CR in the Rochester cohort. Statistical significance was assessed using the two-sided Wilcoxon rank-sum test. **(E)** Schematic of clinical cohort. **(F)** IFITM3 H-scores across patients with PD, SD, or PR. Representative IHC images show IFITM3, MHC-I, and CD8 staining in PR vs. PD tumors (scale bar: 40 μm). Statistical significance was assessed using the two-sided Wilcoxon rank-sum test. **(G)** Scatter plot showing correlations between IFITM3 and MHC-I, as well as between IFITM3 and CD8 H-scores. Spearman correlation was used to assess statistical significance. **(H)** HRs with 95% confidence intervals for PFS among patients with high versus low IFITM3 expression, defined by increasing H-score percentile cutoffs. A range of thresholds (from 15–90%) was tested in the IHC-stained clinical cohort (*n* = 42). HRs and P values were calculated using Cox proportional hazards regression models. **(I)** Kaplan-Meier survival curve for PFS among the indicated groups, with a 75% H-score cutoff for IFITM3 and a 55% cutoff for MHC-I. Survival differences were assessed using the log-rank test. *P < 0.05, **P < 0.01, n.s., not significant

Given the importance of MHC-I molecules in antitumor immunity, we explored whether IFITM3 expression was correlated with immunotherapy efficacy in SCLC. Using the IPS algorithm, we evaluated the immune landscape of SCLC tumors, including MHC molecules, effector immune cells (ECs), suppressive immune cells (SCs), and immune checkpoints (ICs), in two SCLC datasets (sclc_ucologne_2015 and GSE60052). *IFITM3* expression was significantly associated with increased effector immune cell activity and reduced inhibitory immune cell activity (Fig. 3A). Furthermore, immune cell profiling via the MCP-counter algorithm demonstrated a strong positive correlation between *IFITM3* expression and both CD8^+^ T cells and cytotoxic lymphocytes across multiple datasets (Supplementary Fig. 4A), suggesting that IFITM3 may play a pivotal role in antitumor immunity and the response to T-cell-dependent immunotherapy.

To validate this, we analyzed RNA-seq data from two publicly available immunotherapy cohorts and one in-house cohort, including the IMpower133 cohort (116 patients treated with chemotherapy and anti-PD-L1 therapy) [23], the Rochester cohort (29 patients treated with PD-1 inhibitors) [18], and our phase II trial cohort (39 patients treated with chemotherapy and anti-PD-1 therapy) [17]. Across all three cohorts, we observed consistent and significant positive correlations between *IFITM3* expression, key MHC-I genes, and effector T-cell infiltration (Supplementary Fig. 4B-C).

Importantly, high *IFITM3* expression was associated with a significant improvement in PFS in chemoimmunotherapy-treated patients but was correlated with poorer prognosis in the chemotherapy-only groups in the IMpower133 cohort (Fig. 3B). Similarly, in another chemotherapy-treated cohort, elevated *IFITM3* expression was associated with a trend toward shorter PFS (Supplementary Fig. 4D). These findings indicate that patients with elevated IFITM3 expression are more likely to benefit from immunotherapy than from chemotherapy. Our phase II trial cohort reinforced this observation, showing a PFS advantage in patients with high *IFITM3* expression who received chemoimmunotherapy (Fig. 3C), whereas PD-1 responders exhibited higher IFITM3 expression in the Rochester cohort (Fig. 3D).

To further assess the clinical relevance of IFITM3, we performed IHC analysis of tumor samples from 42 patients treated with chemotherapy and PD-1/PD-L1 inhibitors (Fig. 3E, Supplementary Table 3). IFITM3 expression was the highest in patients with a PR, intermediate in those with stable disease (SD), and lowest in those with progressive disease (PD) (Fig. 3F). IHC staining for MHC-I and CD8 markers further confirmed a significant positive correlation between IFITM3 expression, MHC-I expression, and CD8^+^ T-cell infiltration (Fig. 3G). To define the optimal cutoff for IFITM3 stratification, we evaluated a range of H-score thresholds. The results indicated that higher IFITM3 cutoff scores were associated with progressively lower disease risk. Among these, the 75th percentile (H-score = 115) demonstrated the strongest association with improved PFS (Fig. 3H). Accordingly, we defined IFITM3-high cases as those with H-scores above the 75th percentile (H-score > 115). Although MHC-I also showed some predictive ability, its effect was weaker and less statistically significant across various H-scores than that of IFITM3 (Supplementary Fig. 4E). Next, we evaluated whether a combination of IFITM3 and MHC-I staining would offer better predictive value. We used the 55th percentile as the cutoff for MHC-I expression and categorized the patients into three groups: high coexpression of IFITM3 and MHC-I, high expression of either marker, and low expression of both markers. Survival analysis revealed the longest PFS in patients with high coexpression and the shortest PFS in those with low expression of both markers (Fig. 3I). These findings establish IFITM3 as a reliable biomarker for predicting immunotherapy outcomes in SCLC patients. Furthermore, the practicality of IFITM3 IHC staining makes it a valuable tool for screening SCLC patients in clinical settings. Combined assessment of IFITM3 and MHC-I expression levels may further improve the predictive accuracy of immunotherapy efficacy.

Given that first-line treatment for SCLC typically includes chemotherapy, and that corticosteroids are commonly used in clinical practice and have been reported to impair antitumor immune responses, particularly at high doses [32–34], we investigated whether these treatments affect IFITM3 expression. SCLC cell lines were treated with low and high doses of cisplatin or dexamethasone for 24 and 48 h, respectively. Western blot analysis showed that IFITM3 protein levels remained unchanged following treatment with either cisplatin (Supplementary Fig. 4F) or dexamethasone (Supplementary Fig. 4G). These results suggest that chemotherapy and corticosteroid exposure do not significantly impact IFITM3 expression, supporting the stability of IFITM3-based patient stratification and its potential utility as a predictive biomarker under standard clinical conditions.

### IFITM3 sensitizes SCLC tumors to PD-1 inhibitors

**Fig. 4:**
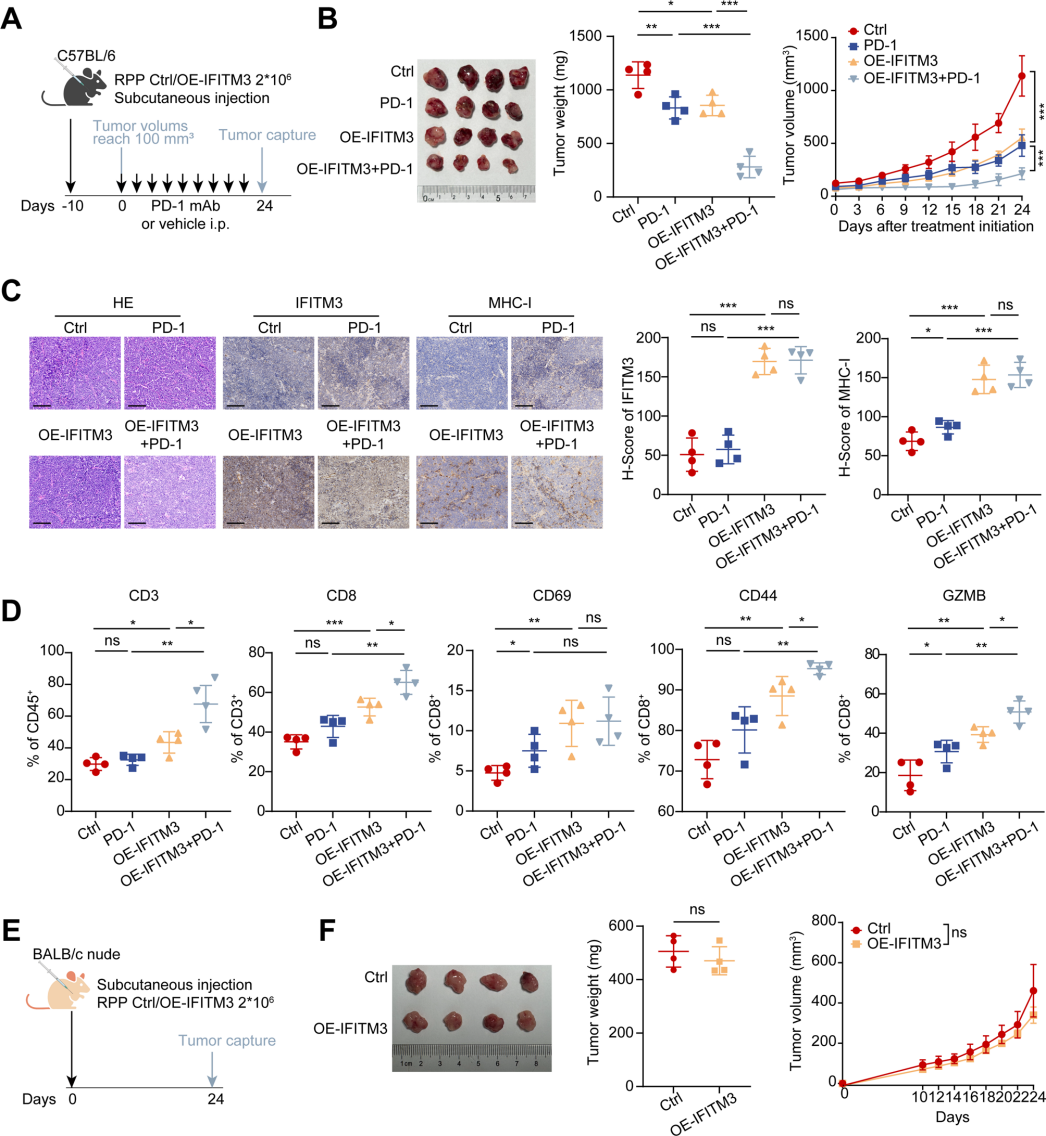
IFITM3 enhances PD-1 inhibitor efficacy and tumor immune responses in SCLC mouse models. **(A)** A schematic view of the treatment plan. C57BL/6 mice were injected with RPP control or OE-IFITM3 cells, and a PD-1 mAb or vehicle was administered when the tumors reached approximately 100 mm³. **(B)** Representative images and weight plots of tumors, along with tumor volume plots measured every 3 days. **(C)** H&E staining and IFITM3 and MHC-I IHC analyses of tumor sections from the indicated treatment groups (scale bar: 100 μm). **(D)** Flow cytometry analysis of CD3^+^ CD45^+^ cells, CD8^+^ CD3^+^ cells, and CD69^+^, CD44^+^, and GZMB^+^ CD8^+^ cells. **(E)** A schematic view of the treatment plan. BALB/c nude mice were injected with RPP-control or OE-IFITM3 cells. **(F)** Representative images and weight plots of tumors harvested after the mice were euthanized, along with tumor volume plots measured every 2 days. The results are presented as the means ± SEMs, *n* = 4. Statistical comparisons between groups were performed using unpaired two-tailed Student’s t-tests. *P < 0.05, **P < 0.01, ***P < 0.001, n.s., not significant

The pronounced response to PD-1/L1 therapy observed in patients with elevated IFITM3 expression underscores the potential of IFITM3 induction to sensitize SCLC tumors to immunotherapy. To test this hypothesis, *Rb1*^−/−^, *p53*^−/−^, and *p130*^−/−^ (RPP) murine SCLC cells stably expressing either a control vector or IFITM3 (Supplementary Fig. 5A) were subcutaneously transplanted into immunocompetent C57BL/6 mice. Anti-PD-1 treatment was initiated once the average tumor volume reached approximately 100 mm³ (Fig. 4A). Compared with the control, IFITM3 overexpression significantly inhibited tumor growth, and the addition of anti-PD-1 further enhanced tumor suppression (Fig. 4B). IHC analysis confirmed that IFITM3-overexpressing tumors presented significantly elevated MHC-I protein levels (Fig. 4C).

To determine the effects of IFITM3 alone and in combination with anti-PD-1, we conducted multiplex immune profiling of the tumor-infiltrating immune cell subsets (Supplementary Fig. 5B). We observed that IFITM3-overexpressing tumors exhibited significantly increased infiltration of both CD3⁺ and CD8⁺ T cells, and these levels were further elevated when combined with anti-PD-1 therapy, suggesting a broader activation of T cell-mediated immune responses. Additionally, these tumors presented elevated levels of CD69 + early activated T cells, CD44 + effector T cells, and granzyme B + cytotoxic CD8^+^ T cells, demonstrating that IFITM3 enhances both CD8^+^ T-cell infiltration and cytotoxic activity (Fig. 4D). To determine whether the antitumor effects of IFITM3 overexpression were T-cell dependent, RPP cells expressing either a control vector or IFITM3 were transplanted into nude mice lacking mature T-cell function (Fig. 4E). In this T-cell-deficient model, tumor growth in IFITM3-overexpressing mice was comparable to that observed in the controls, indicating that the antitumor effects of IFITM3 overexpression are dependent on T-cell-mediated immunity (Fig. 4F).

Collectively, these findings demonstrate that IFITM3 overexpression triggered a potent antitumor immune response and sensitized SCLC tumors to PD-1 blockade, highlighting its potential as a therapeutic target for immunotherapy.

### EG sensitizes SCLC to PD-1 inhibitors by inducing IFITM3 expression

**Fig. 5:**
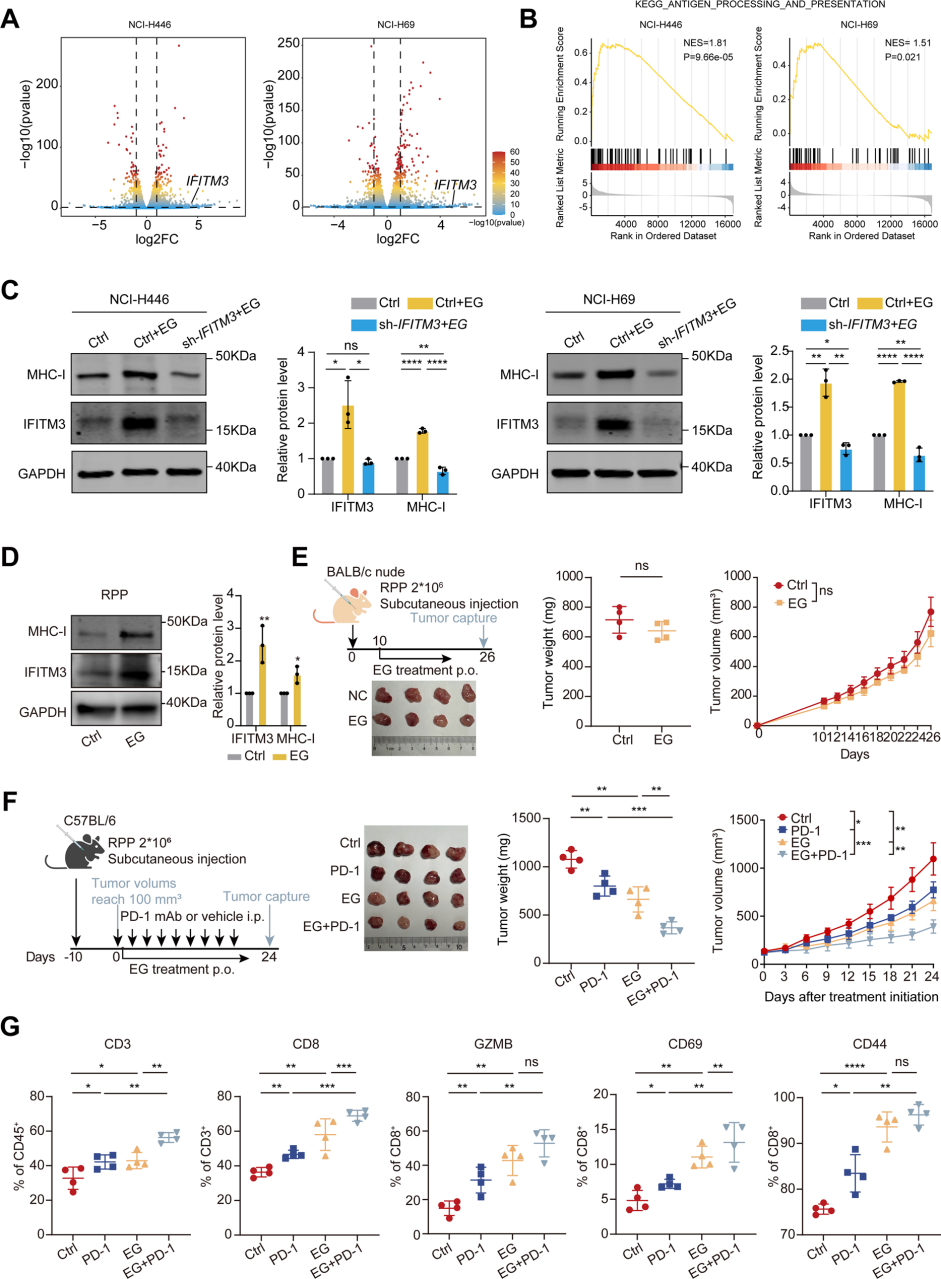
EG enhances the efficacy of PD-1 inhibition and antitumor immune responses in SCLC mouse models. **(A)** Volcano plots showing differential gene expression following EG treatment in NCI-H446 and NCI-H69 cells. *IFITM3* is significantly upregulated upon EG treatment. Vertical dashed lines indicate log2FC = ± 1, and the horizontal dashed line represents the significance threshold at *P* = 0.05. **(B)** GSEA plots for the KEGG_ANTIGEN_PROCESSING_AND_PRESENTATION pathway in EG-treated NCI-H446 and NCI-H69 cells. NES and P values are shown. **(C)** Western blot analysis of IFITM3 and MHC-I protein levels in NCI-H446 and NCI-H69 cells treated with EG or transduced with sh-IFITM3, as indicated. Densitometric quantification is shown. **(D)** Western blot analysis of IFITM3 and MHC-I protein levels in RPP cells treated with EG (40 μm) for 48 h. **(E)** Schematic of the BALB/c nude mouse model for subcutaneous injection of RPP cells followed by oral EG treatment. Representative images and weight plots of tumors, along with tumor volume plots measured every 2 days. **(F)** C57BL/6 mice were implanted with RPP cells and received EG and PD-1 mAb treatment. Representative images and weight plots of tumors along with tumor volume plots measured every 3 days. **(G)** Flow cytometry analysis of CD3^+^ CD45^+^ cells, CD8^+^ CD3^+^ cells, and CD69^+^, CD44^+^, and GZMB^+^ CD8^+^ cells. The results are presented as the means ± SEMs, *n* = 4. Statistical comparisons between groups were performed using unpaired two-tailed Student’s t-tests. Statistical comparisons between groups were performed using unpaired two-tailed Student’s t-tests. *P < 0.05, **P < 0.01, ***P < 0.001, ****P < 0.0001, n.s., not significant

EG, a phenolic compound derived from walnut kernels, *Euphorbia fischeriana*, and *Galla rhois*, possesses potent antioxidant and anticancer properties [35]. Previous studies have reported that EG upregulates IFITM3 [36]. In our study, we found that EG induced IFITM3 expression in both a dose- and time-dependent manner (Supplementary Fig. 6A-B). To further assess the transcriptomic effects of EG in SCLC, we performed RNA sequencing on EG-treated and control H446 and H69 cells. PCA revealed clear separation between EG-treated and control samples, indicating distinct transcriptomic profiles (Supplementary Fig. 6C). Differential expression analysis showed significant upregulation of *IFITM3* and multiple MHC-I related genes following EG treatment (Fig. 5A, Supplementary Table 4). GSEA consistently demonstrated significant enrichment of the antigen processing and presentation pathway in both cell lines (Fig. 5B), whereas other immune-related pathways were not consistently enriched (Supplementary Fig. 6D-E). Notably, apart from *IFITM3* and MHC-I associated genes, the majority of ISGs showed minimal or no changes in expression (Supplementary Fig. 6F), suggesting that EG selectively enhances IFITM3-mediated MHC-I antigen presentation without broadly activating interferon signaling.

Furthermore, Western blot analysis confirmed that EG treatment markedly increased both IFITM3 and MHC-I expression in control cells, whereas this effect was largely abolished in IFITM3-knockdown cells (Fig. 5C), indicating that the immunomodulatory activity of EG is largely dependent on IFITM3.

EG treatment also induced IFITM3 and MHC-I expression in RPP cells (Fig. 5D). In line with the observations from the IFITM3 overexpression studies, EG treatment alone did not significantly inhibit tumor growth in nude mice (Fig. 5E). To investigate whether EG enhances the sensitivity of SCLC cells to anti-PD-1 therapy, tumor-bearing mice were treated with anti-PD-1, anti-EG, or a combination of both. EG treatment alone significantly reduced tumor size and weight, and its combination with anti-PD-1 therapy further amplified tumor suppression (Fig. 5F). Throughout the treatment course, no significant body weight loss or overt signs of toxicity were observed in EG-treated mice, indicating good in vivo tolerability (Supplementary Fig. 6G). Flow cytometry analysis demonstrated that EG markedly increased CD3 + T-cell and CD8^+^ T-cell infiltration and increased the proportions of CD69 + early activated T cells, CD44 + effector T cells, and granzyme B + cytotoxic CD8^+^ T cells, mirroring the immune cell changes observed with IFITM3 overexpression (Fig. 5G). These findings indicate that EG potentiates antitumor immunity by upregulating IFITM3 and enhancing MHC-I expression, thus positioning it as a promising candidate for combination immunotherapy.

### IFITM3 enhances MHC-I expression via NLRC5 upregulation and nuclear trafficking

**Fig. 6:**
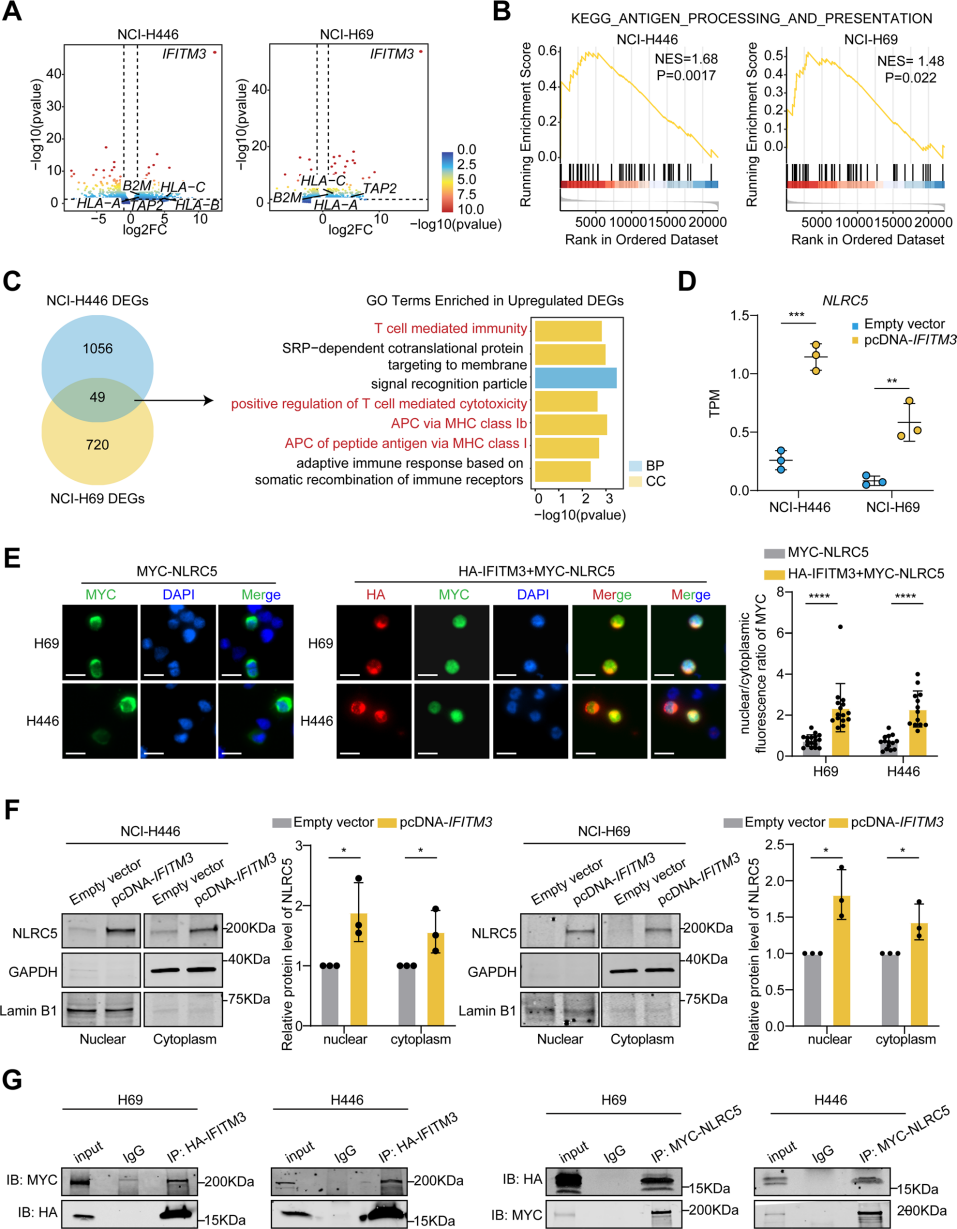
IFITM3 enhances MHC-I expression via NLRC5 upregulation and nuclear trafficking in SCLC cells. **(A)** Volcano plots showing DEGs between IFITM3-overexpressing NCI-H446 and NCI-H69 cells and control cells. Vertical dashed lines indicate log2FC = ± 1, and the horizontal dashed line represents the significance threshold at *P* = 0.05. **(B)** GSEA showing activated antigen processing and presentation pathways caused by IFITM3 overexpression in NCI-H446 and NCI-H69 cells. Enrichment significance was determined using the NES and nominal P values, as calculated by the GSEA algorithm. **(C)** Venn diagram of overlapping DEGs between NCI-H446 and NCI-H69 cells, accompanied by a bar plot of enriched GO terms. **(D)** Box plots displaying *NLRC5* expression levels in NCI-H446 and NCI-H69 cells following *IFITM3* overexpression. **(E)** Immunofluorescence images of NCI-H69 and NCI-H446 cells overexpressing MYC-NLRC5 (green) and/or HA-IFITM3 (red). Nuclei were counterstained with DAPI (blue). Scale bar: 20 μm. **(F)** Subcellular fractionation assays showing NLRC5 expression in cytoplasmic and nuclear fractions following IFITM3 overexpression. **(G)** Co-IP of IFITM3 with NLRC5 in NCI-H446 and NCI-H69 cells. The results are presented as the means ± SEMs. Statistical comparisons between groups were performed using unpaired two-tailed Student’s t-tests. *P < 0.05, **P < 0.01, ***P < 0.001, ****P < 0.0001

To investigate the mechanism by which IFITM3 regulates MHC-I expression, RNA sequencing was performed in NCI-H446 and NCI-H69 cells overexpressing IFITM3. PCA revealed clear separation between IFITM3-overexpressing and control cells (Supplementary Fig. 7A). Notably, IFITM3 overexpression significantly upregulated key MHC-I related genes, including *HLA-A*, *HLA-B*, *HLA-C*, *TAP2*, and *B2M* (Fig. 6A). GSEA revealed pronounced enrichment of the antigen presentation pathway in IFITM3-overexpressing cells (Fig. 6B). In contrast, neither the interferon-α nor interferon-γ response gene sets were consistently enriched in either cell line (Supplementary Fig. 7B-C). Only a limited number of ISGs were significantly upregulated following IFITM3 overexpression, while the majority of ISGs remained unchanged (Supplementary Fig. 7D). These findings suggest that IFITM3 enhances MHC-I expression without triggering a global interferon response, supporting a model in which IFITM3 regulates MHC-I through a more specific mechanism.

To further delineate this mechanism, we performed differential gene expression analysis (Supplementary Table 5) and identified 49 commonly upregulated genes in both SCLC cell lines. GO enrichment analysis showed that these genes were significantly enriched in pathways related to MHC-I mediated antigen presentation (Fig. 6C). Among these genes, *NLRC5* has emerged as a critical regulator of MHC-I (Fig. 6D), which is consistent with its established role as a transcriptional activator of MHC-I and the antigen-processing machinery [37]. Notably, EG treatment also significantly upregulated *NLRC5* (Supplementary Fig. 8A). Analysis of multiple SCLC datasets revealed a significant positive correlation between *IFITM3* and *NLRC5* expression (Supplementary Fig. 8B). Clinically, higher *NLRC5* expression was associated with prolonged PFS in the chemoimmunotherapy cohorts, while the opposite trend was observed in patients receiving chemotherapy alone (Supplementary Fig. 8C-D). RT-qPCR and western blotting analyses confirmed that *IFITM3* overexpression significantly increased *NLRC5* mRNA and protein levels (Supplementary Fig. 8E-F). These findings indicate that IFITM3 may enhance MHC-I expression by modulating NLRC5, thereby contributing to improved sensitivity to PD-1/PD-L1 blockade in SCLC.

Previous studies have highlighted the critical role of IFITM3 in intracellular trafficking and the ability of NLRC5 to shuttle between the cytoplasm and nucleus to regulate MHC-I expression [38]. On the basis of these findings, we hypothesized that IFITM3 influences NLRC5 intracellular trafficking and thereby regulates MHC-I expression. IF analysis revealed that NLRC5 was localized primarily to the cytoplasm in NCI-H69 and NCI-H446 cells. Notably, IFITM3 overexpression enhanced the nuclear translocation of NLRC5 (Fig. 6E). Consistently, subcellular fractionation assays revealed that IFITM3 overexpression increased NLRC5 levels in both the cytoplasmic and nuclear fractions (Fig. 6F), suggesting that IFITM3 not only promotes NLRC5 expression but also facilitates its nuclear localization. Co-IP assays further confirmed a direct interaction between IFITM3 and NLRC5, supporting the hypothesis that this interaction facilitates NLRC5 nuclear translocation (Fig. 6G). These findings establish that IFITM3 upregulates MHC-I expression by promoting NLRC5 expression and facilitating its nuclear trafficking, thereby enhancing antigen presentation and improving immunotherapy efficacy in SCLC.

## Discussion

MHC-I downregulation is a key immune evasion mechanism in SCLC, which significantly limits the efficacy of PD-1/PD-L1 inhibitors. In tumor cells, alterations in the MHC-I pathway can arise through both genetic and epigenetic mechanisms, affecting various components of the MHC-I antigen processing and presentation machinery (APM) [39]. While tumors of various histological subtypes often acquire genetic alterations in the MHC-I APM as an adaptive response to immune pressure during immunotherapy, SCLC is inherently characterized by intrinsically low MHC-I levels. Recent evidence suggests that this deficiency is partly driven by epigenetic programming and that targeting epigenetic regulators, such as EZH2, LSD1, and MEN1, can induce MHC-I in SCLC [4, 10–12]. In this study, we identified IFITM3 for the first time as a regulator of MHC-I expression in SCLC cells. IFITM3 promotes the expression of the MHC-I transcriptional regulator *NLRC5* and facilitates its nuclear trafficking, enriching our understanding of the MHC-I regulatory mechanisms in SCLC.

Previous studies have linked elevated IFITM3 expression to tumor progression in glioblastoma, gastric, and hepatocellular carcinomas, where it regulates key pathways, such as cell proliferation, angiogenesis, and epithelial-mesenchymal transition (EMT) [40–42]. Notably, IFITM3 has also been shown to modulate the tumor microenvironment by promoting CD8^+^ T-cell infiltration, which is essential for antitumor immunity [43], whereas its deficiency disrupts antigen processing and presentation in cervical cancer cells [44, 45]. These findings highlight the versatile and context-specific role of IFITM3 in various malignancies. Our study demonstrated that IFITM3 upregulates MHC-I expression and enhances tumor sensitivity to PD-1 inhibitors in SCLC patients by promoting CD8 + T cell infiltration and cytotoxic. In addition, IFITM3 overexpression significantly increased CD3⁺ total T-cell infiltration within the tumor microenvironment. These findings suggest that IFITM3 may not only facilitate the recruitment of cytotoxic CD8⁺ T cells but also contribute to the establishment of a broader T cell-inflamed phenotype. The observed expansion of total T cells further supports the role of IFITM3 in reshaping the immune landscape of SCLC toward a more immunoresponsive state.

Through transcriptome analysis, we found that IFITM3 induced *NLRC5*, a key transcription factor of MHC-I. Given that NLRC5 has been reported to shuttle between the cytoplasm and nucleus in HeLa cells [38] and that IFITM3 has been shown to facilitate the trafficking of incoming viral particles to lysosomes for degradation to inhibit viral infection [46, 47], we further investigated the possibility that IFITM3 mediates NLRC5 trafficking in SCLC cells. Our findings demonstrated that IFITM3 interacts with NLRC5 and promotes its translocation from the cytoplasm to the nucleus, thereby inducing MHC-I expression. Overall, our study revealed a novel mechanism by which IFITM3 regulates antitumor immunity.

Furthermore, our integrative analysis of real-world SCLC cohorts demonstrated that patients with high *IFITM3* expression had significantly longer PFS after treatment with anti-PD-1 inhibitors. In contrast, *IFITM3* expression showed no notable benefit in patients receiving chemotherapy, highlighting its relevance to immune-based therapeutic strategies. Previously, both PD-L1 expression and the TMB have failed to correlate with treatment efficacy in this setting. The findings from our in-house cohort suggest that IFITM3 IHC staining could serve as a potential biomarker for predicting immunotherapy response, potentially outperforming MHC-I expression alone. Importantly, our study revealed that combining IFITM3 and MHC-I expression further improved the predictive accuracy, suggesting that this dual-marker approach could offer a more comprehensive predictor of immunotherapy outcomes in SCLC patients. We also demonstrated that IFITM3 expression is not affected by standard therapeutic agents such as cisplatin or dexamethasone, supporting its stability and reliability as a predictive biomarker and reinforcing its potential clinical utility for pre-treatment patient stratification.

Finally, our identification of EG as an inducer of IFITM3 presents a promising therapeutic strategy. Although the anticancer properties of EG have been previously established [48, 49], our findings reveal its role in modulating the immune microenvironment. EG significantly upregulated IFITM3 and MHC-I expression in SCLC cell lines and enhanced PD-1 blockade efficacy in vivo. EG-treated tumors presented increased CD8^+^ T-cell infiltration and activation, recapitulating the immune effects observed with IFITM3 overexpression. These results suggest that EG could be a valuable combination therapy with ICIs, particularly for SCLC patients with low MHC-I expression, and warrants further investigation in the clinical setting.

This study has several limitations and areas that merit further investigation. First, although IFITM3 emerged as a promising predictive biomarker for immunotherapy in SCLC, its clinical utility requires validation in larger, independent patient cohorts. In addition, further studies are needed to establish clinically actionable thresholds for IFITM3 expression to support patient selection and therapeutic decision-making in future clinical settings. Second, beyond SCLC, IFITM3 expression showed significant positive correlations with multiple MHC-I genes in tumors characterized by low baseline MHC-I expression. These findings suggest that IFITM3 may have broader relevance across tumor types and underscore the need for further functional investigations in additional cancer contexts. Third, although our preclinical data suggest that EG may enhance antitumor immunity and synergize with PD-1 blockade, its safety, pharmacokinetics, and therapeutic efficacy in clinical settings remain to be determined through future translational studies.

## Conclusions

**Fig. 7:**
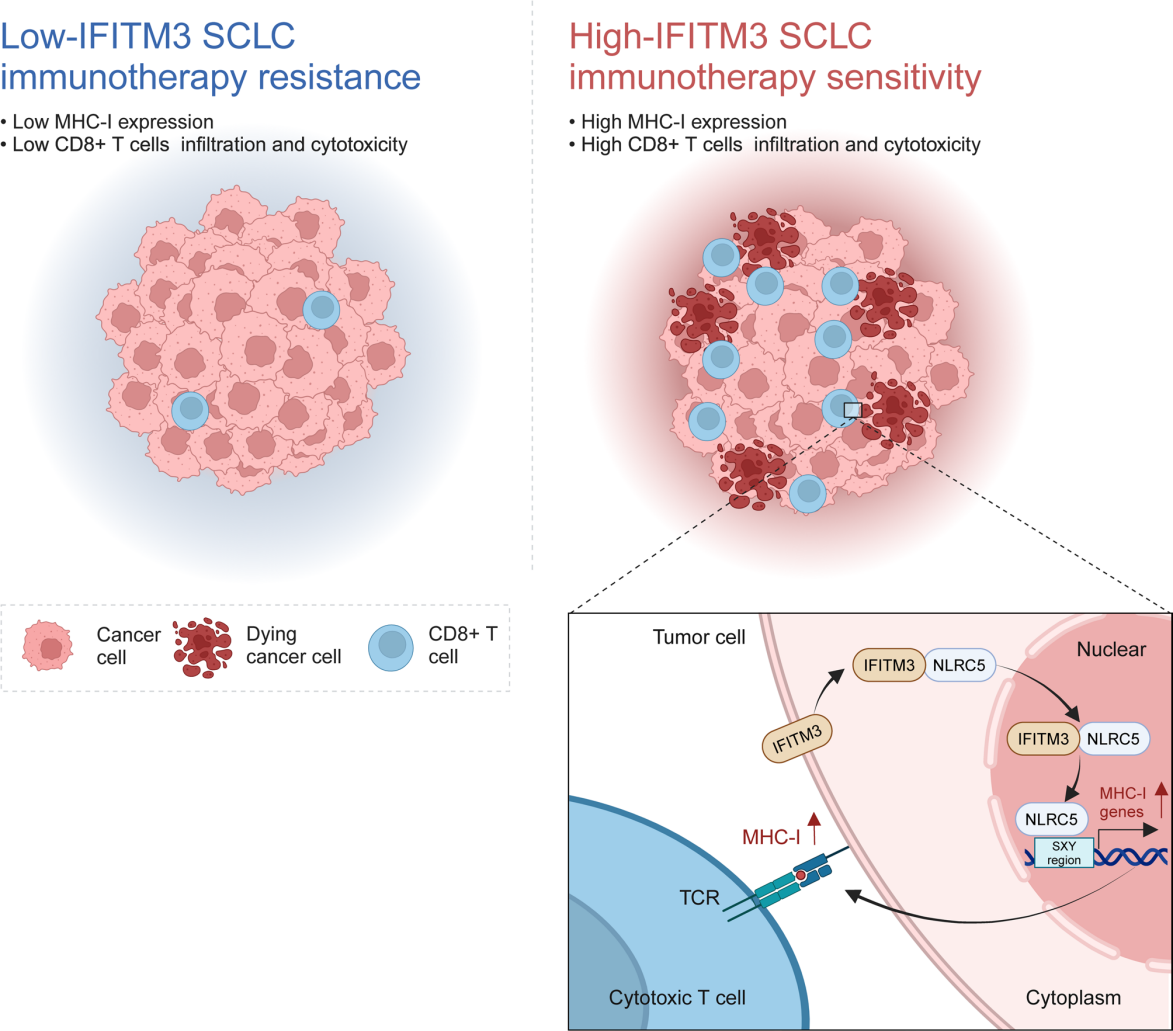
Mechanism of action of IFITM3 in regulating MHC-I expression to enhance immunotherapy sensitivity in SCLC

In summary, we found that IFITM3 could serve as a potent biomarker for predicting immunotherapy efficacy in SCLC and that its overexpression could enhance CD8^+^ T-cell infiltration and promote antitumor immunity. Mechanistically, IFITM3 upregulates NLRC5 and promotes its nuclear translocation, thereby increasing MHC-I transcription. EG, a potent IFITM3 inducer, has the potential to overcome resistance to immunotherapy (Fig. 7). These findings underscore the clinical relevance of IFITM3 staining in SCLC and provide a rationale for further clinical investigations of EG in combination with PD-1 inhibitors in SCLC patients.

## Electronic supplementary material

Below is the link to the electronic supplementary material.

Supplementary Material 1

Supplementary Material 2

Supplementary Material 3

## Data Availability

The datasets used and/or analyzed during the current study are available from the corresponding author upon reasonable request.

## Abbreviations

ICIs: Immune checkpoint inhibitors
SCLC: Small cell lung cancer
TMB: Tumor mutational burden
MHC-I: Major histocompatibility complex class I
PRC2: Polycomb repressive complex 2
LSD1: Lysine-specific demethylase 1
ITH: Intratumoral heterogeneity
scRNA-seq: Single-cell RNA sequencing
IFITM3: Interferon-induced transmembrane protein 3
UMAP: Uniform manifold approximation and projection
GO: Gene Ontology
GEO: Gene Expression Omnibus
CCLE: Cancer Cell Line Encyclopedia
IPS: Immune phenotypes
EGA: European Genome-phenome Archive
PFS: Progression-free survival
FFPE: Formalin-fixed paraffin-embedded
RECIST: Response Evaluation Criteria in Solid Tumors
PR: Partial response
CR: Complete response
DAB: Diaminobenzidine
ORF: Open reading frame
FBS: Fetal bovine serum
GEMM: Genetically engineered mouse model
RPP: Rb family p130
ORF: Open reading frame
RT-qPCR: Reverse Transcription quantitative Polymerase Chain Reaction
RIPA: Radioimmunoprecipitation assay
SDS-PAGE: Sulfate-polyacrylamide gel electrophoresis
ECL: Enhanced chemiluminescence
EG: Ethyl gallate
PCA: Principal component analysis
DEGs: Differentially expressed genes
GSEA: Gene set enrichment analysis
NES: Normalized enrichment scores
ISGs: Interferon-stimulated genes
Co-IP: Coimmunoprecipitation
BSA: Bovine serum albumin
EC: Effector immune cells
SC: Suppressive immune cells
IC: Immune checkpoint inhibitors
SD: Stable disease
PD: Progressive disease
EMT: Epithelial-mesenchymal transition
AUC: Area under the curve
ORR: Objective response rate
HR: Hazard ratio
IHC: Immunohistochemical
LUAD: Lung adenocarcinoma
