# Supplementary material for: IFITM3 enhances immunosensitivity via MHC-I regulation and is associated with the efficacy of anti-PD-1/-L1 therapy in SCLC
Source: Mol Cancer. 2025 Jul 3;24:187. doi: 10.1186/s12943-025-02383-x (PMC12225533; doi:10.1186/s12943-025-02383-x)
Supplement: Supplementary file 1 — Supplementary Material 1 [file 12943_2025_2383_MOESM1_ESM.pdf]

Supplementary Data

IFITM3 enhances immunosensitivity via MHC-I regulation and is associated with the efficacy of anti-PD-1/-L1 therapy in SCLC

Contents

Supplementary Figure 1. Clustering and subtype characterization of single-cell RNA-seq data in SCLC.

Supplementary Figure 2. Analysis of IFITM3 expression and its association with MHC-I related genes across datasets and cancer types**.**

Supplementary Figure 3. IFITM3 regulates MHC-I expression in SCLC cell lines.

Supplementary Figure 4. Association of IFITM3 expression with immune features, MHC-I expression, clinical outcomes, and drug-induced protein changes.

Supplementary Figure 5. Overexpression of IFITM3 enhances MHC-I expression and promotes immune activation in SCLC.

Supplementary Figure 6. EG induces IFITM3 and enhances MHC-I antigen presentation in SCLC cells.

Supplementary Figure 7. Transcriptomic effects of IFITM3 overexpression in SCLC cells.

Supplementary Figure 8. Correlation of IFITM3 with NLRC5 expression and prognostic value of NLRC5 in SCLC.

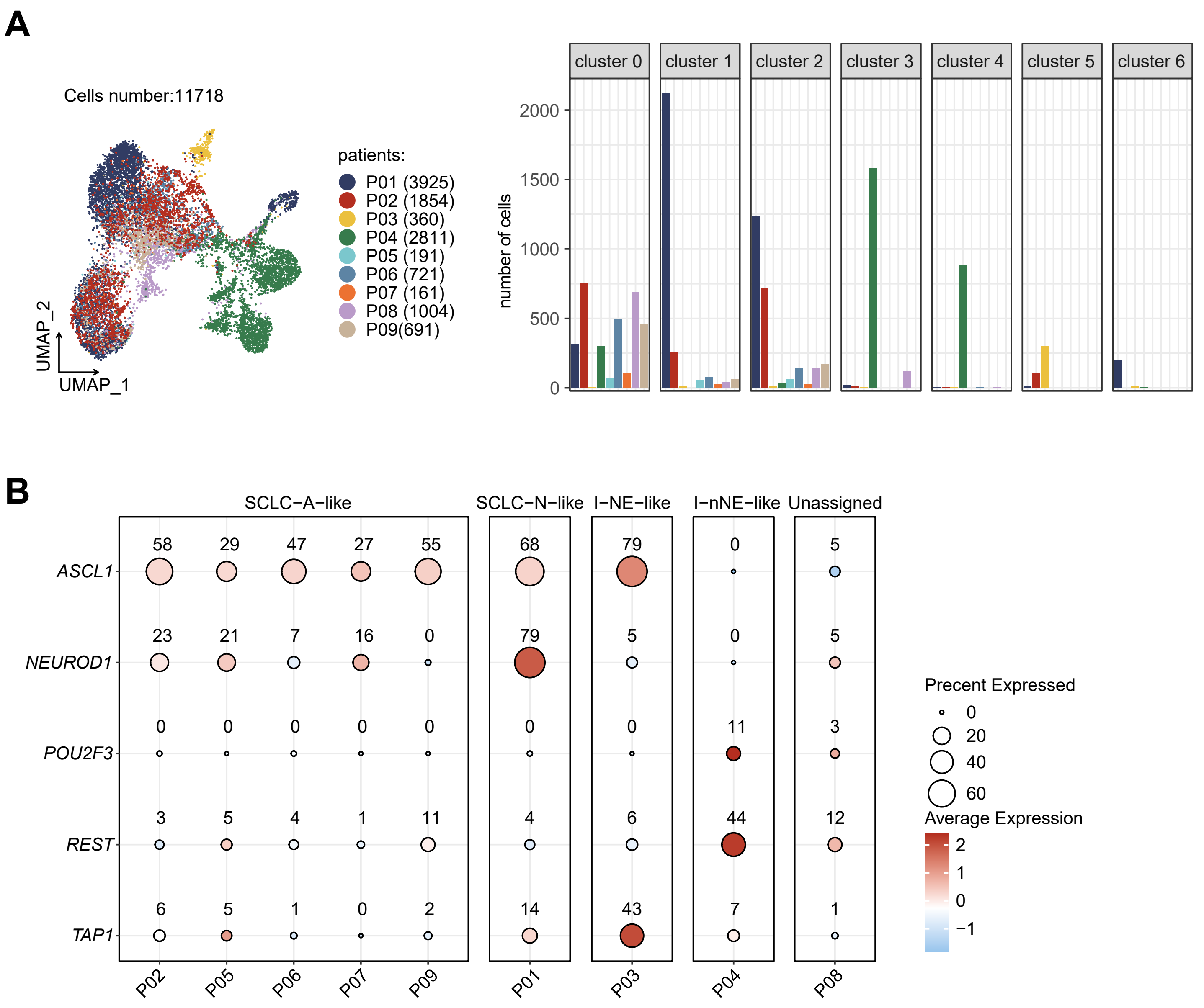

Supplementary Figure 1. Clustering and subtype characterization of single-cell RNA-seq data in SCLC. (A) UMAP plot showing 11,718 cells clustered into distinct subpopulations across different SCLC samples, color-coded by their original identities (left), Bar plots illustrating the distribution of cells from each sample across the seven identified clusters (Cluster 0 to Cluster 6). The number of cells within each cluster is indicated (right). (B) Dot plot depicting the expression profiles of key transcription factors (*ASCL1, NEUROD1, POU2F3*, and *REST*) and the MHC-I molecule *TAP1* across SCLC subtypes (SCLC-A-like, SCLC-N-like, I-NE-like, and unassigned). Dot size represents the percentage of cells expressing the gene, and color intensity reflects average expression levels.

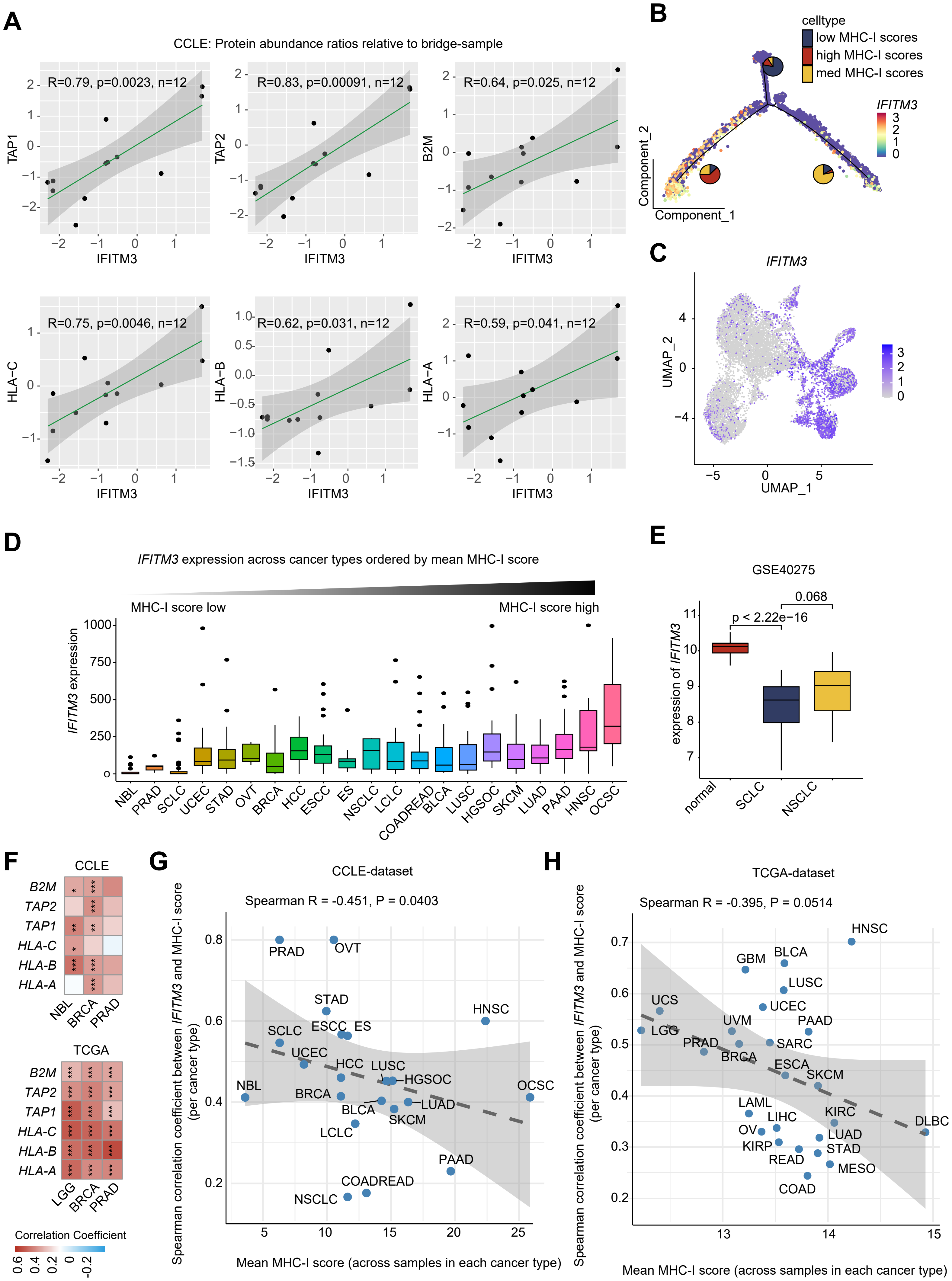

Supplementary Figure 2. Analysis of IFITM3 expression and its association with MHC-I related genes across datasets and cancer types. (A) Scatter plots showing Spearman correlations between IFITM3 protein abundance and selected MHC-I components (TAP1, TAP2, B2M, HLA-C, HLA-B, HLA-A) in the CCLE dataset. Correlation coefficients (R) and p-values are indicated.(B) Pseudotime trajectory analysis of single-cell RNA-seq data, showing *IFITM3* expression levels across cells stratified by MHC-I expression. (C) UMAP plot of single-cell RNA-seq data illustrating the expression distribution of *IFITM3* across different cell populations. (D) *IFITM3* expression levels across cancer types in the CCLE dataset, ranked by the mean MHC-I score. (E) *IFITM3* expression in normal lung, SCLC, and NSCLC samples from the GSE40275 dataset. Statistical comparisons were performed using the Wilcoxon test. (F) Heatmaps showing Spearman correlation coefficients between *IFITM3* and MHC-I-related genes across selected tumor types in the CCLE (top) and TCGA (bottom) datasets. Color scale indicates strength and direction of correlation. (G, H) Scatter plots showing the relationship between the mean MHC-I score (x-axis) and the Spearman correlation coefficient between *IFITM3* and MHC-I score (y-axis) across tumor types in the CCLE (G) and TCGA (H) datasets. Each dot represents one cancer type.

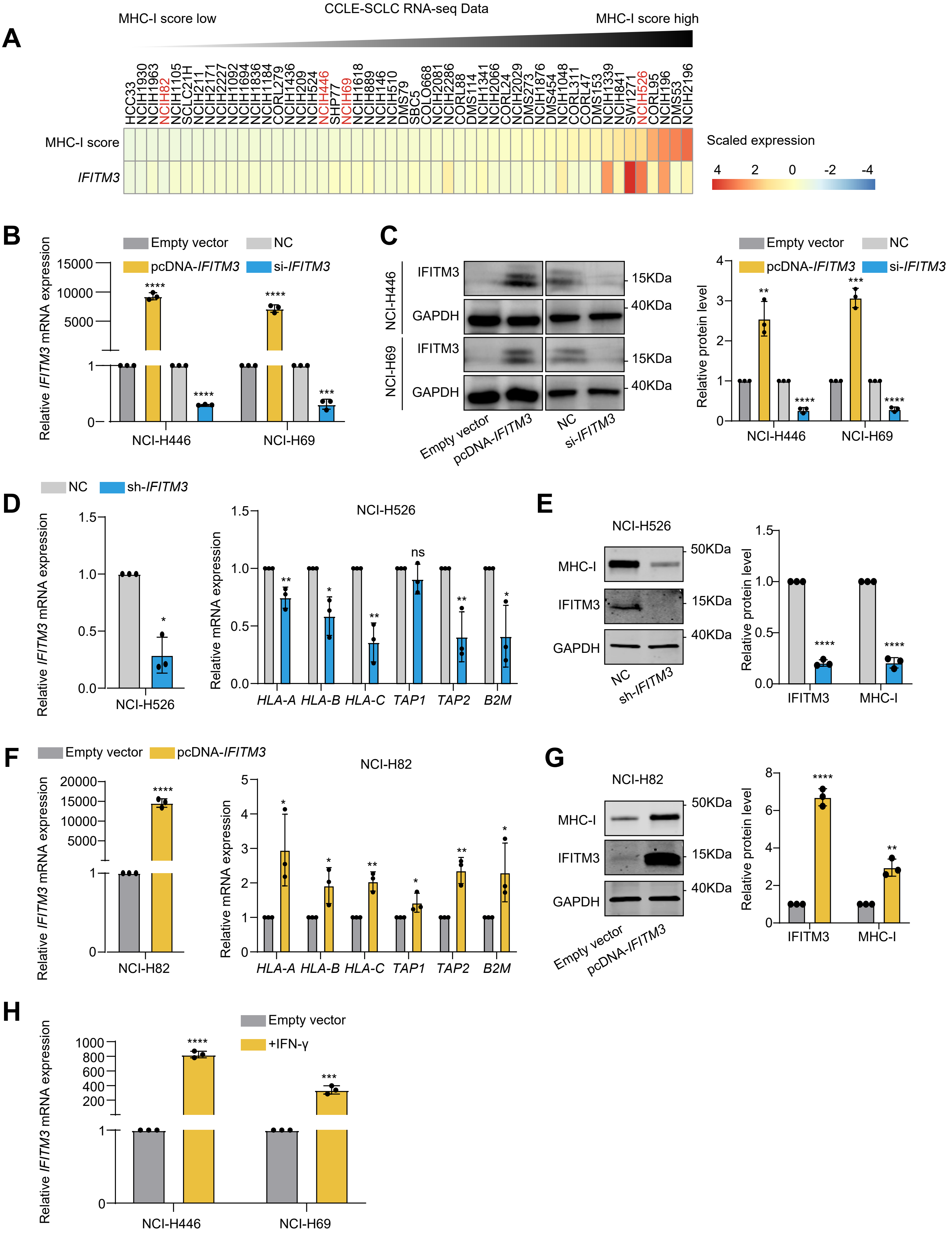

Supplementary Figure 3. IFITM3 regulates MHC-I expression in SCLC cell lines.(A) Heatmap of *IFITM3* expression and MHC-I signature scores across SCLC cell lines from the CCLE RNA-seq dataset. Cell lines selected for functional experiments are highlighted in red. (B) RT-qPCR analysis showing *IFITM3* mRNA levels in NCI-H446 and NCI-H69 cells after *IFITM3* overexpression (pcDNA-*IFITM3*) or knockdown (si-*IFITM3*). (C) Western blot analysis of IFITM3 protein expression in NCI-H446 and NCI-H69 cells following IFITM3 overexpression or knockdown. (D) RT-qPCR analysis of *IFITM3* and MHC-I related genes in NCI-H526 cells after *IFITM3* knockdown. (E) Western blot and quantification of IFITM3 and MHC-I protein levels in NCI-H526 cells following *IFITM3* knockdown. GAPDH was used as loading control in all blots. (F) RT-qPCR analysis of *IFITM3* and key MHC-I pathway genes in NCI-H82 cells following *IFITM3* overexpression. (G) Western blot and quantification of IFITM3 and MHC-I protein levels in NCI-H82 cells upon *IFITM3* overexpression. (H) *IFITM3* mRNA expression in NCI-H446 and NCI-H69 cells following treatment with IFN-γ. Statistical comparisons were performed using unpaired two-tailed Student's t-tests (****$p < 0.0001$; ***$p < 0.001$; **$p < 0.01$; *$p < 0.05$).

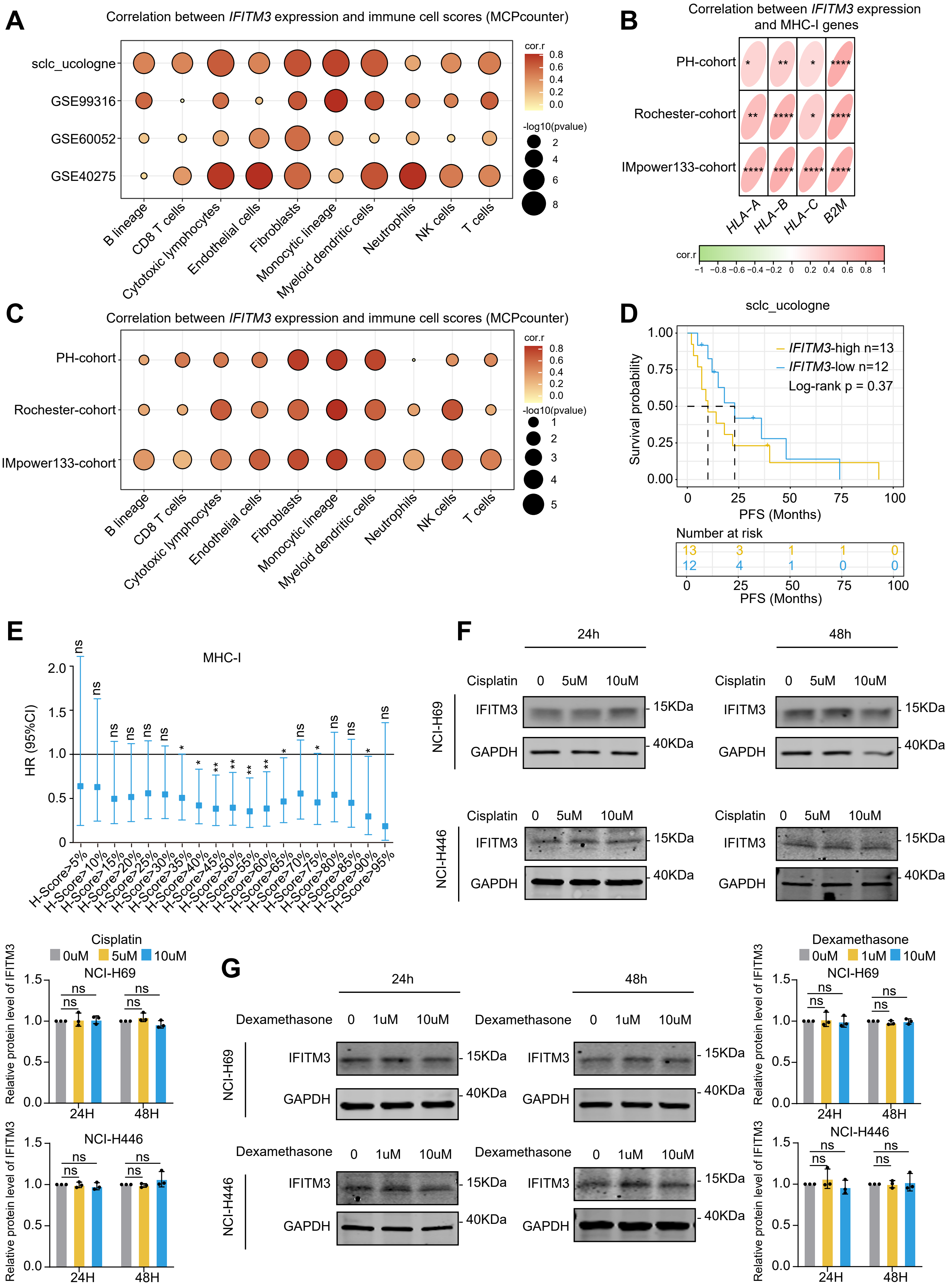

Supplementary Figure 4. Association of IFITM3 expression with immune features, MHC-I expression, clinical outcomes, and drug-induced protein changes. (A) Dot plot showing the correlation between *IFITM3* expression and various immune cell types across four SCLC datasets (sclc_ucologne, GSE993, GSE60052, and GSE40275). Dot size represents the significance (-log10(p-value)), and color intensity reflects the correlation coefficient. (B) Correlation heatmap of *IFITM3* expression with MHC-I molecules in three immunotherapy cohorts (FK, Rochester, and IMpower133). Correlation strength is indicated by color intensity, with significant correlations marked. (C) Dot plot showing the association of *IFITM3* expression with immune cell infiltration across three immunotherapy cohorts. Dot size and color follow the same conventions as in (A). (D) Kaplan-Meier curve comparing PFS between high and low *IFITM3* expression groups in the sclc_ucologne dataset. (E) Hazard ratio (HR) analysis of MHC-I gene expression with PFS across multiple datasets. Bars indicate 95% confidence intervals and marked significant associations. (F) Western blot analysis of IFITM3 protein levels in NCI-H69 and NCI-H82 cells treated with cisplatin at indicated concentrations (0, 5 μM, 10 μM) for 24 and 48 hours.Quantification of relative IFITM3 protein levels normalized to GAPDH is shown below.(G) Western blot analysis of IFITM3 and GAPDH protein levels in NCI-H69 and NCI-H82 cells treated with dexamethasone at indicated concentrations (0, 1 μM, 10 μM) for 24 and 48 hours.Quantification of relative IFITM3 protein levels normalized to GAPDH is shown on the right. (****p < 0.0001, ***p < 0.001, **p < 0.01, *p < 0.05 , ns. not significant).

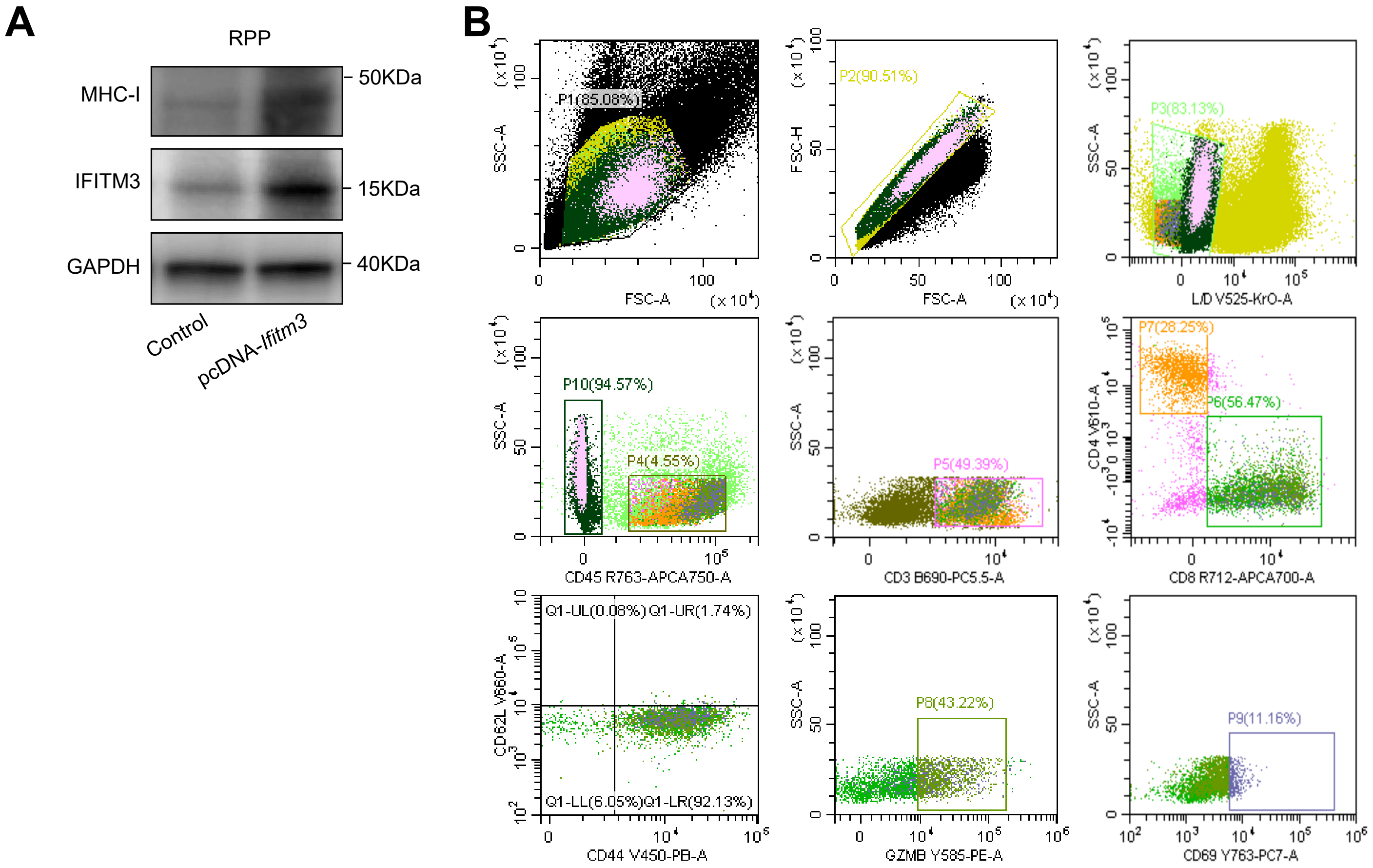

Supplementary Figure 5. Overexpression of IFITM3 enhances MHC-I expression and promotes immune activation in SCLC. (A) Western blot analysis of MHC-I and IFITM3 protein levels in RPP cells transfected with an *Ifitm3* overexpression vector (pcDNA-*Ifitm3*) or control. GAPDH was used as a loading control. (B) Flow cytometry analysis of immune cell populations. Representative gating strategy and percentages of cell subsets are shown.

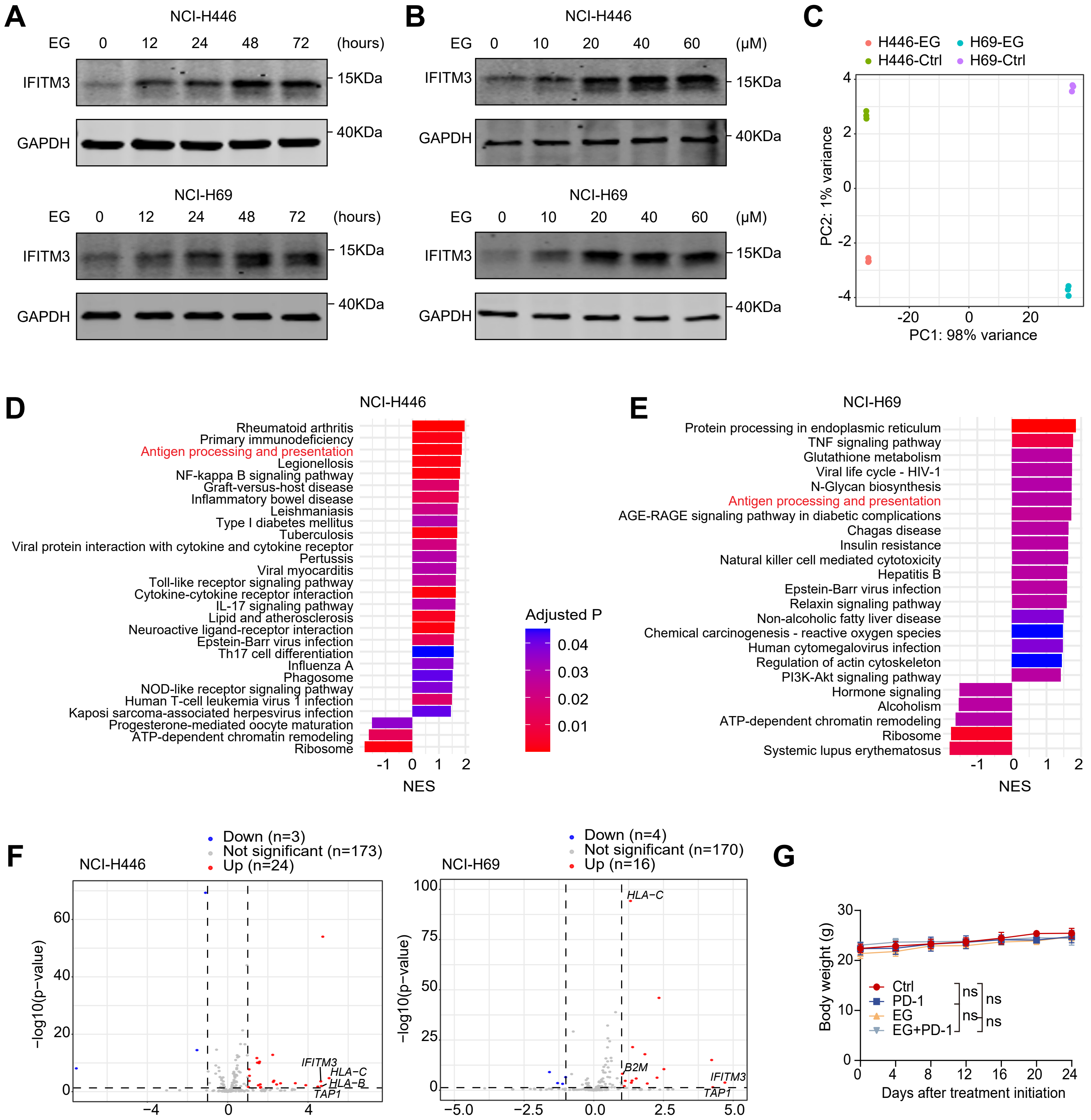

Supplementary Figure 6. EG induces IFITM3 and enhances MHC-I antigen presentation in SCLC cells. (A) Western blot analysis of IFITM3 expression in NCI-H446 and NCI-H69 cells treated with 40 μM EG for the indicated time points (0, 12, 24, 48, and 72 hours). GAPDH was used as a loading control. (B) Western blot analysis of IFITM3 expression in NCI-H446 and NCI-H69 cells treated with increasing concentrations of EG (0, 10, 20, 40, and 60 μM) for 48 hours. (C) PCA based on RNA-seq data reveals clear separation between EG-treated and control samples in both cell lines. (D-E) GSEA of KEGG pathways in EG-treated NCI-H446 (D) and NCI-H69 (E) cells. Pathways with adjusted $p < 0.05$ are shown. The antigen processing and presentation pathway was consistently enriched in both models. (F) Volcano plots showing differentially expressed ISGs in EG-treated NCI-H446 and NCI-H69 cells. *IFITM3* and several MHC-I related genes are highlighted.Dotted lines indicate thresholds of statistical significance (P = 0.05) and fold change (|log2(fold change)| = 1). (G) Body weight monitoring of mice treated with control (Ctrl), PD-1 inhibitor alone, EG alone, or the combination of EG and PD-1 inhibitor. No significant differences in body weight were observed between groups at day 24 (ns, not significant).

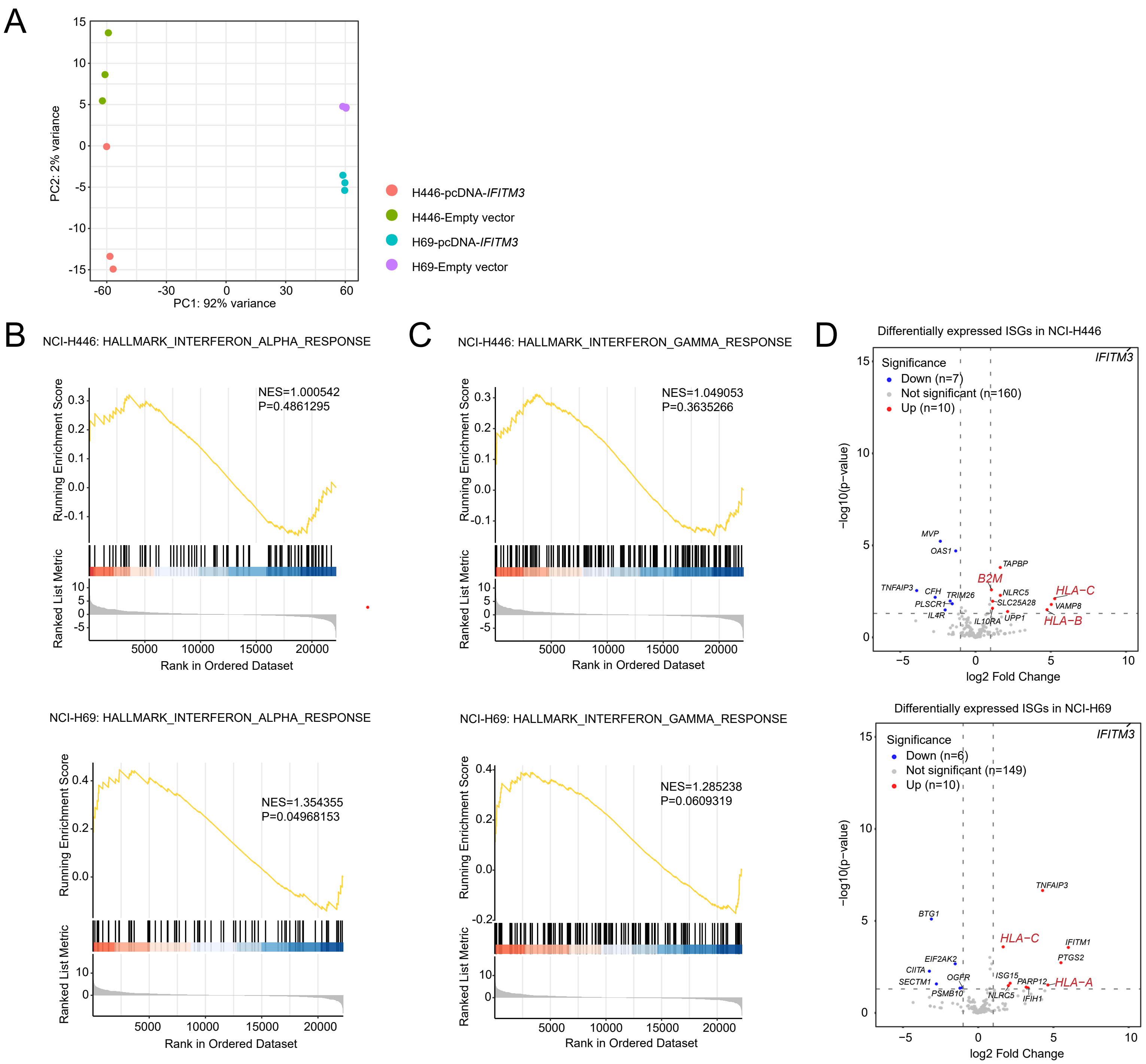

Supplementary Figure 7. Transcriptomic effects of IFITM3 overexpression in SCLC cells. (A) PCA of RNA-seq data from NCI-H446 and NCI-H69 cells transfected with *IFITM3* overexpression plasmid (pcDNA-*IFITM3*) or empty vector. Clear separation was observed between *IFITM3*-overexpressing and control samples. (B–C) GSEA plots of hallmark interferon alpha response (B) and interferon gamma response (C) gene sets in *IFITM3*-overexpressing versus control cells in NCI-H446 (top) and NCI-H69 (bottom). (D) Volcano plots showing differential expression of ISGs in *IFITM3*-overexpressing versus control cells in NCI-H446 (top) and NCI-H69 (bottom). Dotted lines indicate thresholds of statistical significance (P = 0.05) and fold change (|log2(fold change)| = 1).

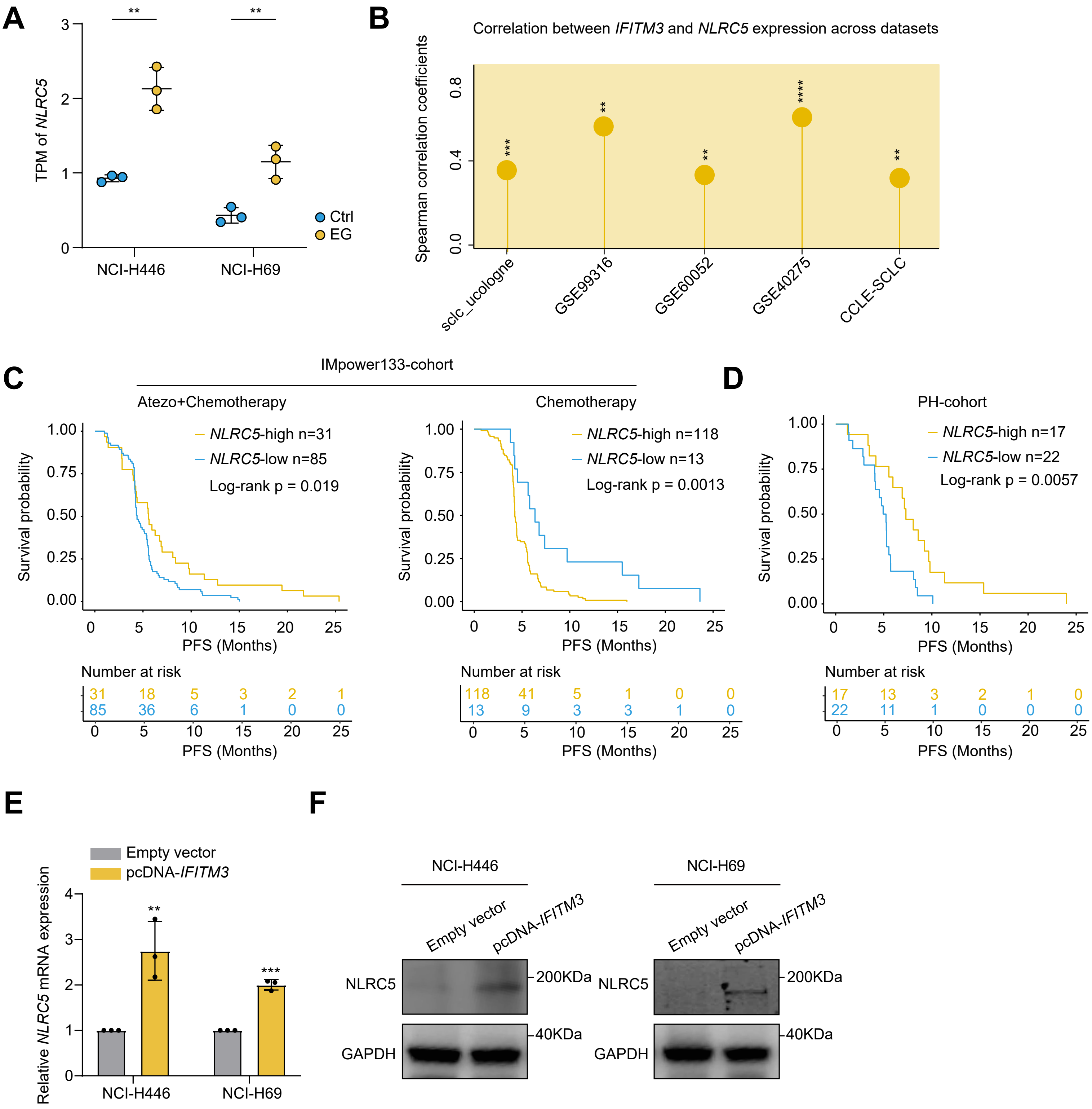

Supplementary Figure 8. Correlation of IFITM3 with NLRC5 expression and prognostic value of NLRC5 in SCLC. (A) *NLRC5* expression levels (TPM) in NCI-H446 and NCI-H69 cells treated with vehicle control (Ctrl) or EG. (B) Spearman correlation coefficients between *IFITM3* and *NLRC5* expression are shown across five datasets. Yellow dots represent the correlation coefficients. (C) Kaplan-Meier survival curves for PFS in the IMpower133 cohort, stratified by the optimal cutoff for *NLRC5* expression. (D) Kaplan-Meier survival curve for PFS in the PH cohort, stratified by optimal cutoff for *NLRC5* expression. (E) qRT-PCR analysis of *NLRC5* mRNA levels in NCI-H446 and NCI-H69 cells following IFITM3 overexpression. Statistical comparisons were performed using unpaired two-tailed Student's t-tests. (F) Western blot analysis of *NLRC5* protein levels in NCI-H446 and NCI-H69 cells following *IFITM3* overexpression. (****p<0.0001, ***p < 0.001, **p < 0.01)
