# Supplementary figures and images for: IFITM3 enhances immunosensitivity via MHC-I regulation and is associated with the efficacy of anti-PD-1/-L1 therapy in SCLC
Source: Mol Cancer. 2025 Jul 3;24:187. doi: 10.1186/s12943-025-02383-x (PMC12225533; doi:10.1186/s12943-025-02383-x)

Fig. 2B

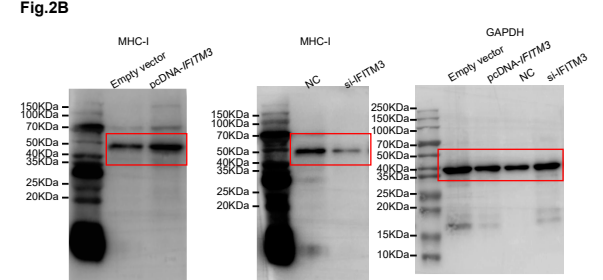

Fig 3C

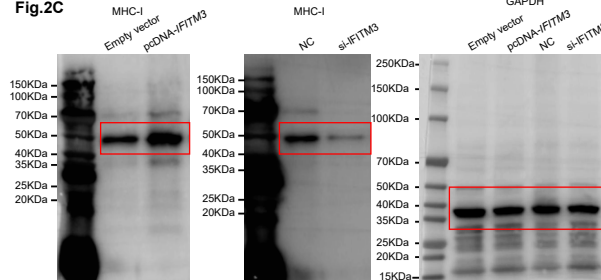

**Fig.5C**

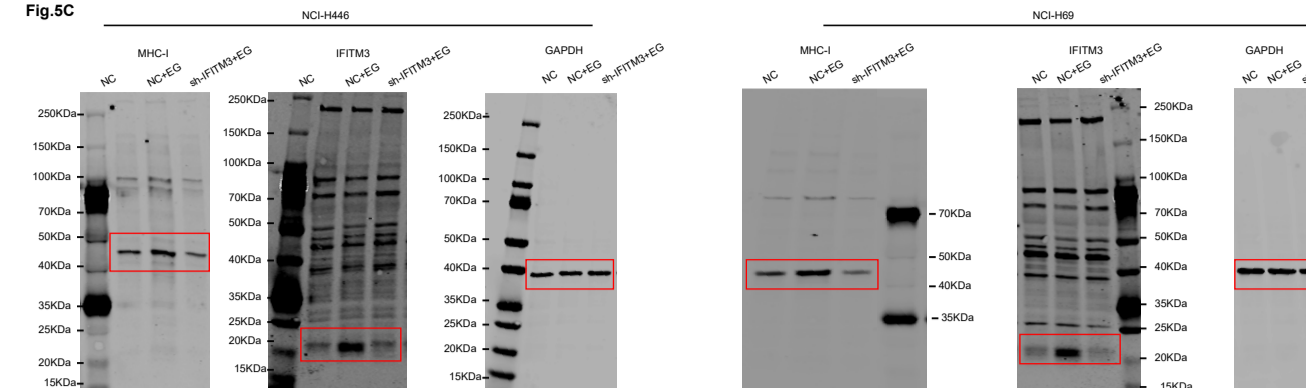

Fig.5D

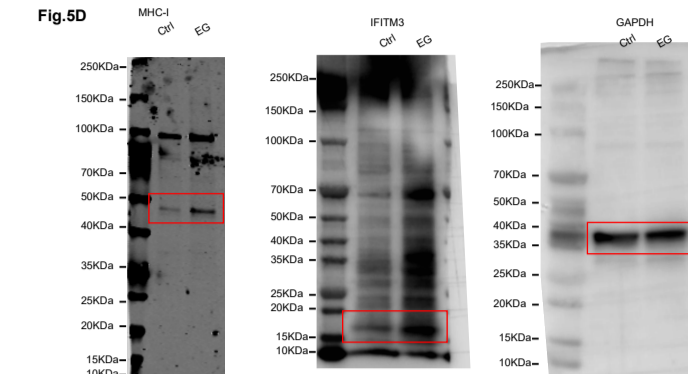

F

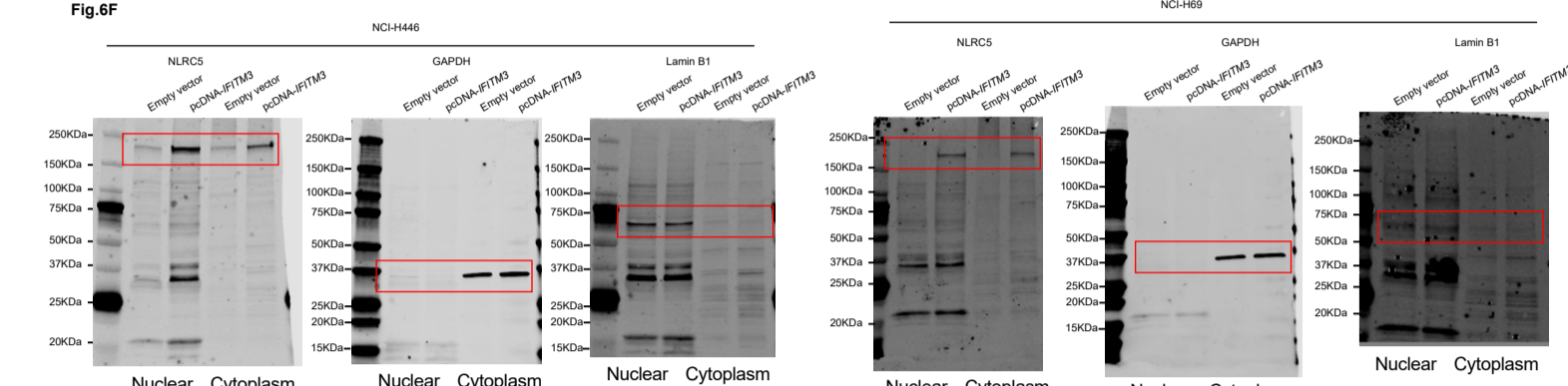

**Fig.6G**

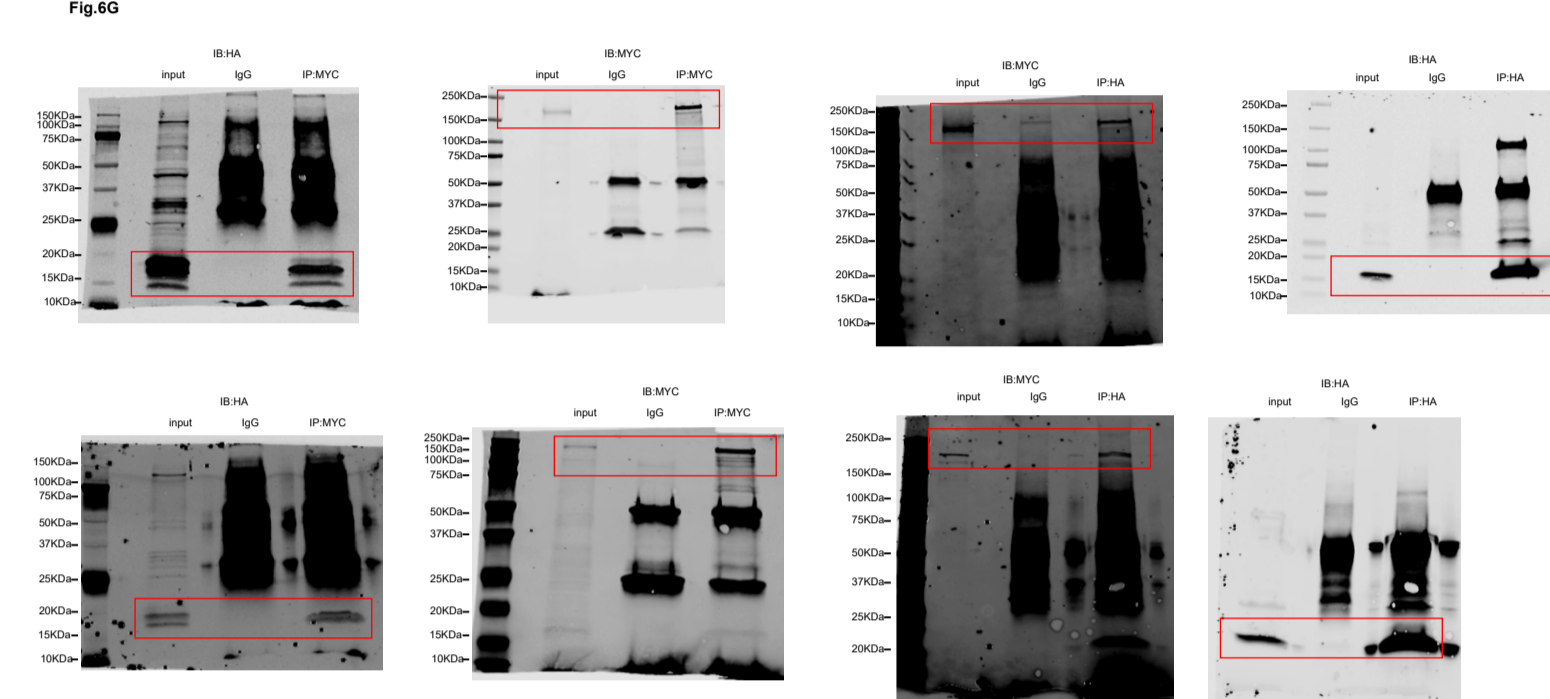

**Fig.S3C**

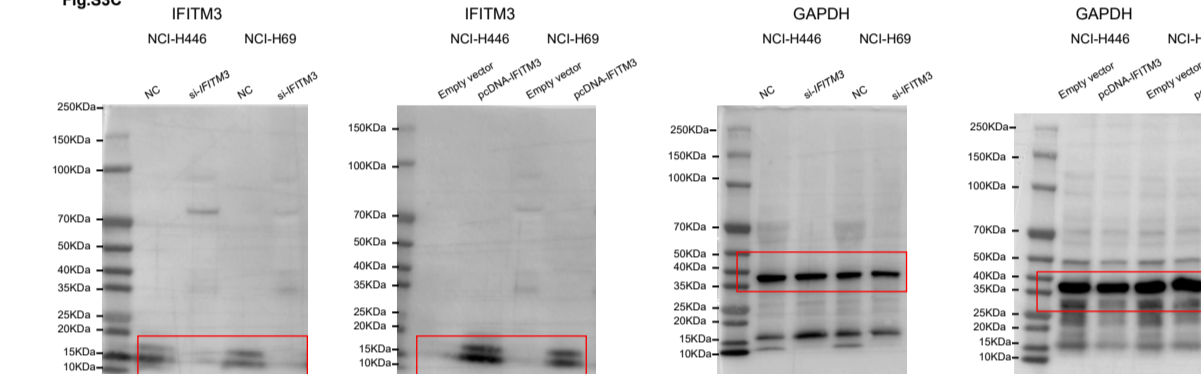

Fig. S25

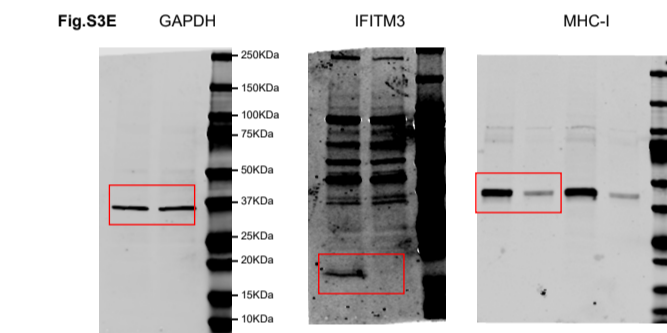

**Fig.S30**

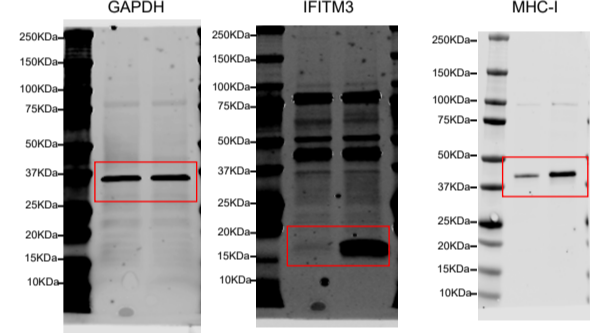

**Fig.S4F**

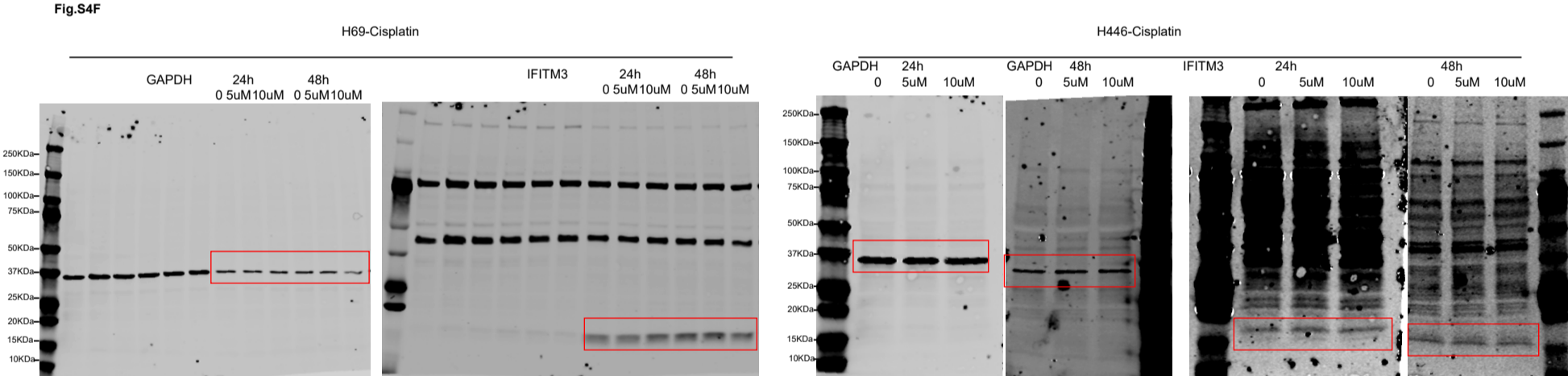

**Fig.S4G**

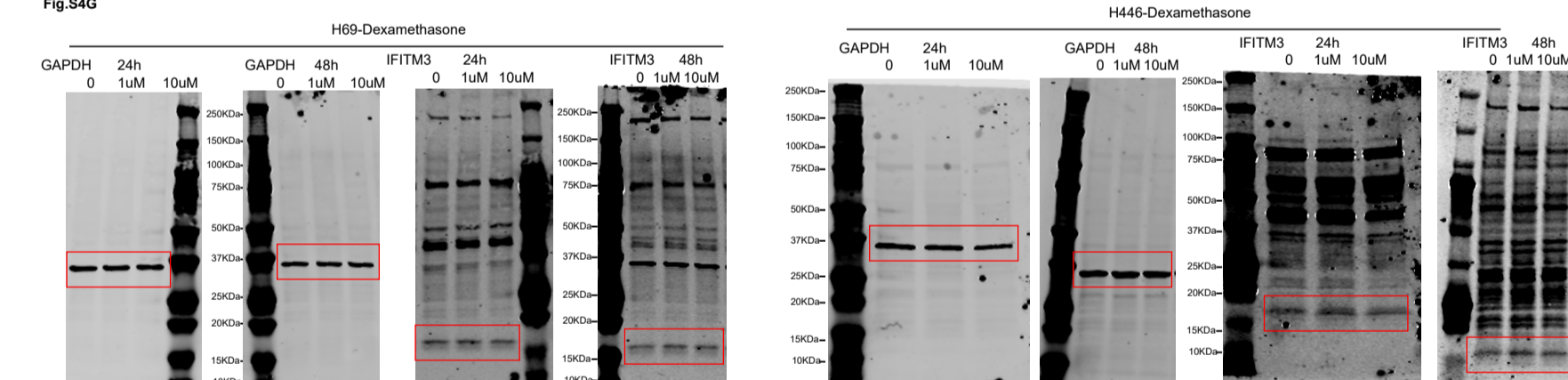

**Fig.S5A**

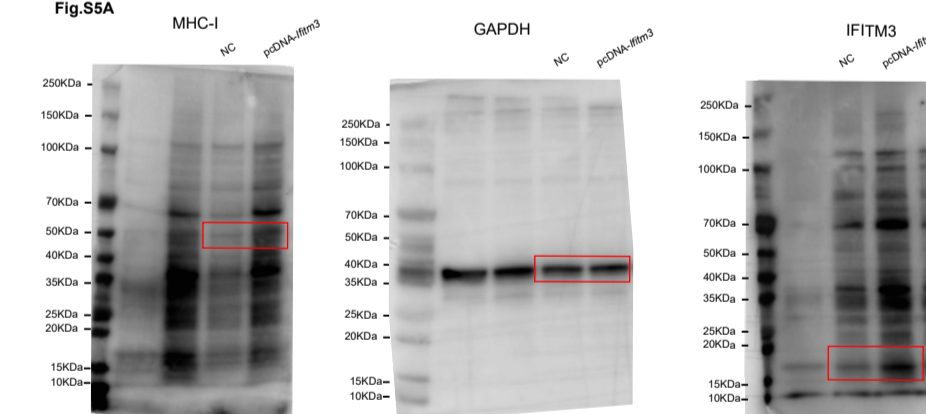

**Fig.S6A**

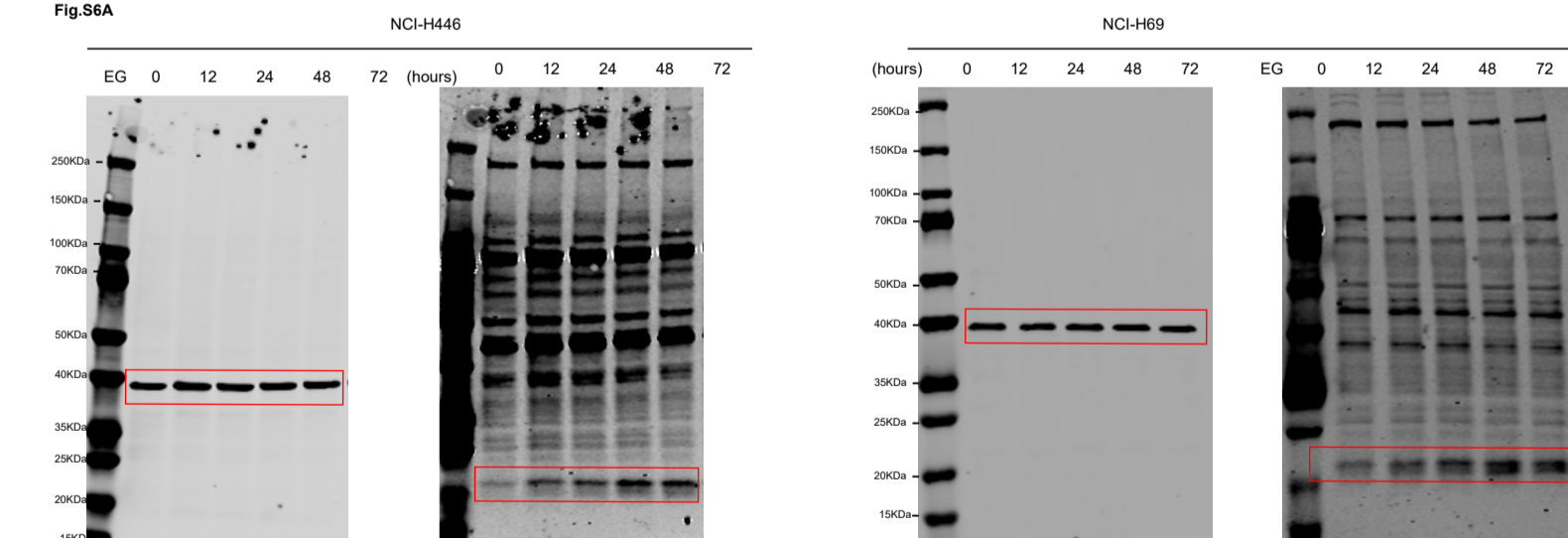

Fig. S6B

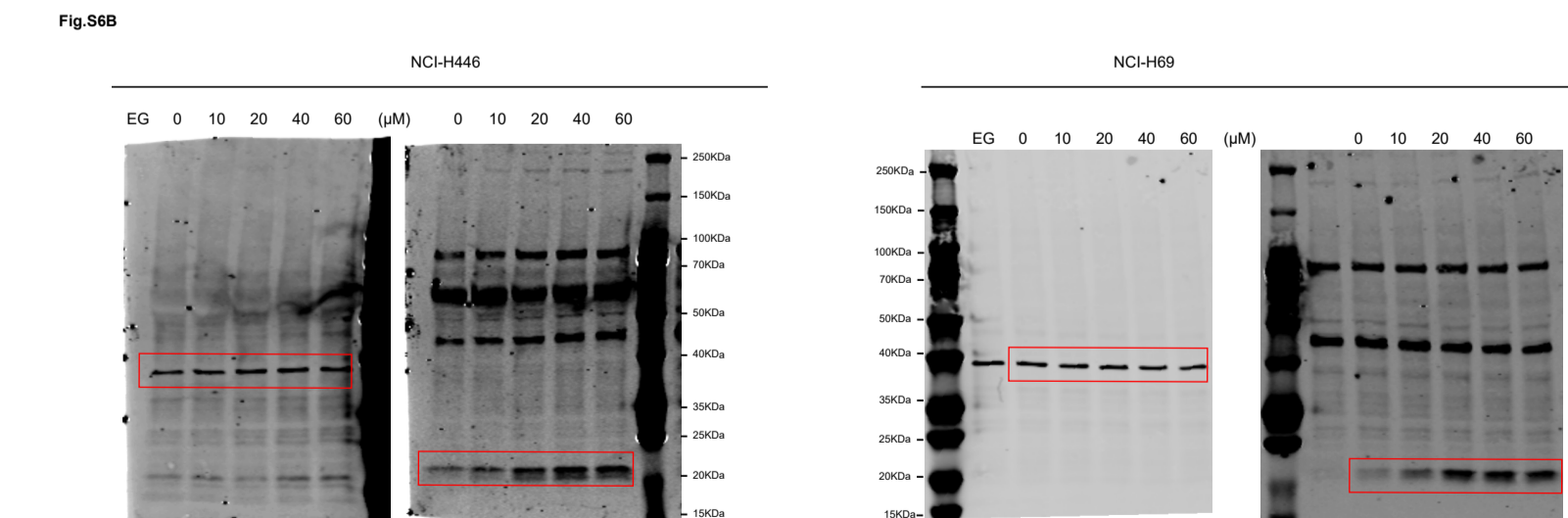

51 22

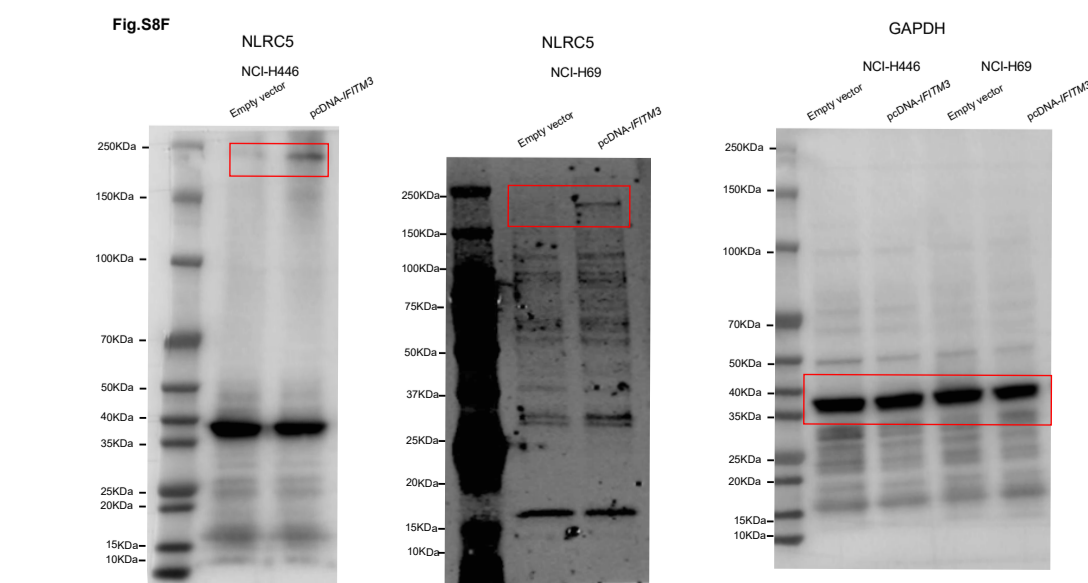

Supplement: Supplementary file 2 — Supplementary Material 2 [file 12943_2025_2383_MOESM2_ESM.pdf]
